# Structural and mechanical properties of humidity-responsive Geraniaceae awns

**DOI:** 10.1038/s41598-025-09186-6

**Published:** 2025-07-01

**Authors:** Marilena Ronzan, Stefano Mariani, Luca Cecchini, Carlo Filippeschi, Silvia Dante, Nicola Maria Pugno, Barbara Mazzolai

**Affiliations:** 1 Bioinspired Soft Robotics Laboratory, Istituto Italiano di Tecnologia, Via Morego 30, Genova, 16163 Italy; 2 Materials Characterization Facility, Istituto Italiano di Tecnologia, Via Morego 30, Genova, 16163 Italy; 3 Laboratory for Bioinspired, Bionic, Meta Materials and Mechanics, Department of Civil, Environmental and Mechanical Engineering, University of Trento, Via Mesiano 77, Trento, 38123 Italy; 4 School of Engineering and Materials Science, Queen Mary University of London, Mile End Road, London, E1 4NS UK

**Keywords:** Composition, Biomechanics, Awn, Dispersion mechanisms, *Erodium*, *Pelargonium.*, Plant sciences, Structural biology

## Abstract

Hygroscopic deformations in plants are passive movements within specialized structures triggered by changes in environmental humidity. In the Geraniaceae, the sterile extension of the mericarp, called awn, facilitates seed dispersal by actuating hygroscopic coiling. Notably, the morphological characteristics and regional distribution of awns vary significantly among the family species, suggesting different mechanisms at the base of dispersion. Despite these variations, no prior investigation has solely focused on examining the combination of the structural and mechanical properties of the awn. Thus, this study fills the gap by conducting an in-depth comparative analysis of the awns from two Geraniaceae species, *Pelargonium appendiculatum* (L.f.) Willd. and *Erodium gruinum* (L.) L’Her., which exhibit similar coiling behavior but possess distinct structural features. Through an interdisciplinary approach, we have identified key internal structural characteristics that profoundly impact the awn’s mechanical properties and hygroscopic response, directly influencing its movement. Our comprehensive findings highlight distinct dispersion mechanisms tailored to each species, providing new insights into the functional role of awn structures. This study not only advances our understanding of plant biomechanics but also highlights the intricate relationship between structure and function.

**Supplementary Information:**

The online version contains supplementary material available at 10.1038/s41598-025-09186-6.

## Introduction

As sessile organisms, plants have evolved various strategies to facilitate seed dispersal and improve germination success (e.g., ballistic ejection, wind, animal-mediated dispersal and hygroscopic movements) though anatomical and morphological adaptations^1^. Hygroscopic movements consist in passive deformations in specific desiccated plant tissues that occur in response to changes in water content. Thanks to the force generated by the contraction of the tissues, which extent depends on the structure, seeds can be released or launched and move across and into soil for germination^2^. The Geraniaceae family, which comprises six genera: *Geranium*,* Hypseocharis (subfam. Hypseocharitoideae)*,* Pelargonium*,* Monsonia*,* Erodium*,* California*, widely distributed from southern Africa to the northern hemisphere^3,4^ is known for the diversity of its seed discharge, also driven by hygroscopic movements. These movements occur through the sterile extension of the mericarp, known as the awn, which serves as a seed dispersal unit and is attached to the encapsulated seed. The awn can deform by bending, coiling or both movements combined, in response to changes in environmental humidity^5^. The morphology of the awns varies significantly within the family and is considered responsible for different dispersal mechanisms. The most ancestral type is the coiling awn, found in both *Erodium* and *Pelargonium*, which likely inherited this trait from a common ancestor. In both genera, the coiling awn aligns to the axis of the mericarp body. Despite this similarity, their structural features and distribution differ, with *Pelargonium* restrained in South Africa and *Erodium* mainly in the Mediterranean region^6^. These contrasts suggest an active adaptation of the awns to the environments in which they have evolved, as observed in other species^1^.

Plant cell walls comprise a mix of cellulose microfibrils embedded in a matrix of polysaccharides such as non-crystalline cellulose, pectin, hemicellulose, besides lignin, and structural proteins^7,8^. The coiling of the awns is influenced by the orientation of cellulose microfibrils in the cell wall, described as a cylindrical helix with a tilted axis, known as microfibril angle (MFA)^9,10^. Cellulose microfibrils represent the crystalline component of the cell wall. When water bonded to non-crystalline cellulose, pectin and hemicellulose is lost due to dehydration, the cell wall structure collapses aligning itself with the orientation of the cellulose microfibrils^10,11^. As a result, the awn bends and twists simultaneously with an anti-clock coiling motion^12^. A single layer of coiling cells can induce this deformation^9^ and it has been reproduced artificially with a *Pelargonium* soft robot^14^. Generally, awns consist of bilayers with different microfibril orientations, defined as the inner active coiling layer and the outer resisting one^2,5^.

In the Geraniaceae family, different dispersal modes have been observed, including fruit release through ballistic launch, wind dispersal, soil exploration, and self-burial. Each of these mechanisms has been analyzed in terms of dynamics, kinematics, and aerodynamics, as discussed in detail below.

For an explosive launch to be successful, elastic energy must be stored in the awn while constrained in a straight position during fruit dehiscence. Stiff materials can store more energy than flexible materials, at high desiccation conditions^15^ as seen in *Erodium moschatum* (L.) L’Hér. which launches its seeds up to 85 cm^16,17^.

Once on the ground, humidity variation causes the coiling and uncoiling of the awn, favoring soil exploration and self-burial of the seeds^16,17^. The extent of these behaviors depends on the dynamic and kinematic responses of the awns. *Erodium moschatum’*s awns explore the soil but remain close to the launch site, while its self-burial capacity improves survivorship and biomass development compared to unburied seeds^16^. Self-burial capacity was also observed in *Pelargonium carnosum* (L.) L’Hér. by measuring the extensional force of the uncoiling awn and the torque from the awn tail rotation^31^.

Flight aerodynamics is influenced by drag force, which is affected by the presence of hairs and branching of the mericarp^18^. Drag force can reduce the flight’s terminal velocity to the wind speed levels causing wind dispersal^15^ as suggested for the *Pelargonium* genus^6^.

The studies previously mentioned, however, have focused on examining individual processes. Furthermore, much research has been conducted mostly on understanding the microfibrils orientation causing the coiling behavior, both in *Erodium* and *Pelargonium* awns^5,9,12^ as well as the effects aromatic compounds distribution on hygroscopic movement in the awns of *Erodium*^32^. Despite the information available, a gap remains regarding the contribution of the awn’s anatomical structures and mechanical properties.

In this study, we use a multidisciplinary approach that integrates structural biology and biomechanics to investigate the awns of Geraniaceae, providing new insights into the mechanisms underlying their movement in the absence of metabolism. Specifically, we examined cell type, composition and material stiffness as key structural attributes, alongside mechanical forces, kinematic performances, and aerodynamics of the awns. We focused on the awns of *Pelargonium appendiculatum* (L.f.) Willd. and *Erodium gruinum* (L.) L’Her., viewed here as two distinct case studies given their distinct morphological traits and relative humidity response, but similar coiling. By identifying the intrinsic, species-specific awn properties, we aim to elucidate the biomechanical principles underlying their dispersal and contribute to a broader understanding of the adaptive evolutionary processes in the Geraniaceae family.

## Materials and methods

### Sample Preparation for image analysis

*Pelargonium appendiculatum* (L.f.) Willd. and *Erodium gruinium* (L.) L’Hér. seeds were purchased from Greenmarket di Barbone Valerio, Bergamo Italy. The awns, i.e. sterile extension of the mericarp to which the encapsulated seed is attached, were weighed at dry and wet conditions, and morphologically and structurally characterized in dry and wet conditions by dividing the awn into three regions (Fig.S1), respectively: base region closest to the seed capsule (called P1 for *Pelargonium* and E1 for *Erodium*); transition region (called P2 for *Pelargonium* and E2 for *Erodium*), and coiling region (called P3 for *Pelargonium* and E3 for *Erodium*). Thin sections of the awns from the three regions were prepared using two methods depending on the type of analysis. Paraffin blocks were obtained by following the protocol reported by Hamann et al.^19^ and Technovit ^®^7100 was used to obtain resin blocks. Sections of 10 and 20 μm thickness were cut with a manual microtome (Leica SM2010R, Germany) and observed with white-light and epifluorescence microscope for observations and measurements (Fig. S2A, B), scanning electron microscope (SEM), RAMAN, and environmental scanning electron microscope (ESEM).

###  White light-epifluorescence microscopy and SEM

*Pelargonium* and *Erodium* sections were stained with Toluidine blue O (0.05%, 1.30 min) and rinsed with deionized water. Cellulose was detected using Alcian blue (pH 2.5, 20 min) and fluorescent brightener 28, observed under UV with a DAPI filter (ex. 340–380 nm). Lignin localization was seen via autofluorescence with a FITC filter (ex. 465–495 nm). All sections were examined with white light and epifluorescence microscopy (Nikon Eclipse Ni-U). For SEM imaging, 10 and 20 μm thick sections and awn samples broken by hand in the coiling region (P3 and E3), were gold sputtered and observed with a Zeiss EVO LS10 at 10 kV. The identification of cell types was performed by combining the observations of SEM images in cut and hand-broken sections with the histological analysis with the polychromatic dye Toluidine Blue O, which differentially stains cell wall components. Generally, poly-aromatic substances, such as lignin and tannin, can be detected in green or light blue in fiber-like cells and dark blue in sclereid-like cells. Carboxylated polysaccharides, such as pectin, are colored purple in the cell wall of parenchyma cells^20,21^. Whereas for the cell composition analysis, Alcian blue, was used to detect acidic polysaccharides like pectin and hemicellulose^22^ and Calcofluor white to detect cellulose^21^.

### Nanoindentation test

The three regions of the awn, previously described and illustrated in Fig.S1, were sampled to measure the material stiffness of the inner (I) and outer (II) layer in respect to the coiling by using INano, Nanomechanics Inc. The samples were investigated by applying the Oliver-Pharr method^23^ using a Berkovitch tip (Young’s modulus E = 1141 GPa, Poisson’s ratio *n* = 0.07) and applying a target triangular function load of 5 mN, target depth 300 nm, with an indentation strain rate of 0.2%/s. During the measurements, the samples were kept at constant relative humidity RH = 50% and continuous temperature T = 25 °C.

### Raman microscopy

The Raman imaging was performed using an in Via Renishaw Microscope (Renishaw, UK) with a 785 nm diode-pumped solid-state laser (100 mW). A 1200 lines/mm diffraction grating and a 50x objective were used. Raman light was detected by an air-cooled CCD with a spectral resolution of 1 cm^−1^. Rectangular maps were recorded with 3 μm spatial resolution in both x and y directions at a fixed z displacement. Exposure time was 0.5 s, with at least 80 accumulations per point. Spectra ranged from 3200 to 2400 cm^−1^ and 1700–600 cm^−1^ of Raman shift, with each pixel representing one average spectrum. Cosmic ray removal and polynomial baseline correction were applied, and the sections were placed on CaF_2_ substrates to reduce background signal. Raman spectroscopy was performed on unstained samples that were obtained through paraffin embedding (Fig. 3A, G, L, M). To minimize the risk of measuring paraffin residues within the samples, we followed a dewaxing protocol as suggested by Mian et al.^24^. This allowed us to confirm that the measurements correspond to functional groups associated with cellulose and pectin.

## Environmental scanning electron microscope (ESEM)

Structure modifications of sections and hand-broken samples under different relative humidity (RH) conditions were observed with SEM Zeiss EVO MA10 equipped with Peltier Stage (Deben UK Ltd.) and Extended Pressure (EP) mode. The EP mode has a 100 μm EP aperture and a 500 μm beams leave. In this condition, the pressure ranges from 10 to 3000 Pascals.

### Dynamic test

Dynamic tests measured blocking force (Fb), moment (M), and extensional force (Fe). Blocking force (Fb), force required for awn to return to its coiling state, was measured from wet to dry awns using the setup in Fig. S6B, with the load cell on a glass slide covered with sandpaper. The end of the inactive awn was centered on the slide. Moment (M) was calculated by multiplying the moment force (Fm) by the length of the inactive awn (r). For moment measurement, combination of bending and torsion, the load cell was also on a sandpaper-covered glass slide (Fig. S6C). For extensional force (Fe), seed capsules were detached from the awn, which was fixed orthogonally to sandpaper with glue (Fig. S6D). A 10 g sensitive load cell (Futek LSB200, Japan) was used and calibrated with a balance (Practum 224-1x, Sartorius, Germany) as shown in Fig. S6A. All measurements were taken after applying water aerosol from a 2 cm distance to the dried awn at 30% relative humidity (RH), with forces and momentum sampled at the maximum value (equilibrium) reached in the real time diagrams (Figs. 4 and 5). The number of the tested samples was three (N samples = 3) for *Pelargonium* and for *Erodium*.

## Kinematic test

Angle displacements (θ, °) and deviations from relative humidity (RH) at 30% (θ30, °) were measured using a climatic chamber (CTC256, Memmert GmbH, Germany) at 30 °C, with RH varying by 10% every 10–20 min (Fig. S7, A, B,C, D,E, F,G, H for *Pelargonium* and Fig. S7I, J,K, L,M, N,O, P for *Erodium*, Table S1). Pitch (P), i.e. the spatial period of the windings along the cylinder helix axis, and Radius (R), i.e. half of the cylindrical helix diameter, were also evaluated under the same conditions (Fig. S9A, B,C, D,E, F,G, H for *Pelargonium* and Fig. S10A, B,C, D,E, F,G, H for *Erodium)*. However, the *Pelargonium* data was reproduced and adapted with permission. (Cecchini, L.; Mariani, S.; Ronzan, M.;Mondini, A.; Pugno, N. M.; Mazzolai, B. 4D Printing of Humidity-Driven Seed Inspired Soft Robots. *Adv. Sci.* 2023, 2205146). Copyright 2023, John Wiley and Sons (CC BY 4.0 DEED). Response times (Rt, s) and angular velocity (ω, °/s) were assessed with a rapid RH increase from 30 to 90%. Videos and pictures were captured with a Logitech Brio Stream webcam (Logitech, Switzerland), po, sitioned 10 cm above the awns for angle measurements and laterally for P and R. Values for θ30, P, and R were recorded at equilibrium. Videos and images were processed with Video Editor (Windows 10, Microsoft) and analyzed using ImageJ software^25^. The number of the tested samples was three (N samples = 3) for *Pelargonium* and for *Erodium*.

## Aerodynamic test

Aerodynamic tests measured terminal speed (vt) by dropping *Pelargonium* and *Erodium* from 2 m, with videos recorded at 240 fps using an Apple iPhone 12 Pro Max (USA). The projected surface (A) was estimated from images of the mericarps taken with a Samsung A13 smartphone (1280 × 800 pixels). The image was binarized, and surface area (S) was determined by counting black pixels within a 1 cm scale bar using ImageJ^25^. In the case of *Pelargonium*, we did not include the hairs in the calculation of the projected area.

### Statistical analysis

When indicated, data are reported as mean values based on N replicates, with error bars representing one standard deviation. The number of samples (N) is specified in parentheses. Given the small sample sizes, approximate normality was assumed. Differences in standard deviations were considered when selecting appropriate statistical tests. A one-way ANOVA was applied to the nanoindentation data to compare the stiffness of multiple regions within the awns, while Welch’s t-test was used for biomechanical analyses due to unequal variances and sample sizes. All statistical comparisons were performed at a significance level of *p* = 0.05 using GraphPad Prism 9.0.

## Results

### Morphological analysis

**Fig. 1:**
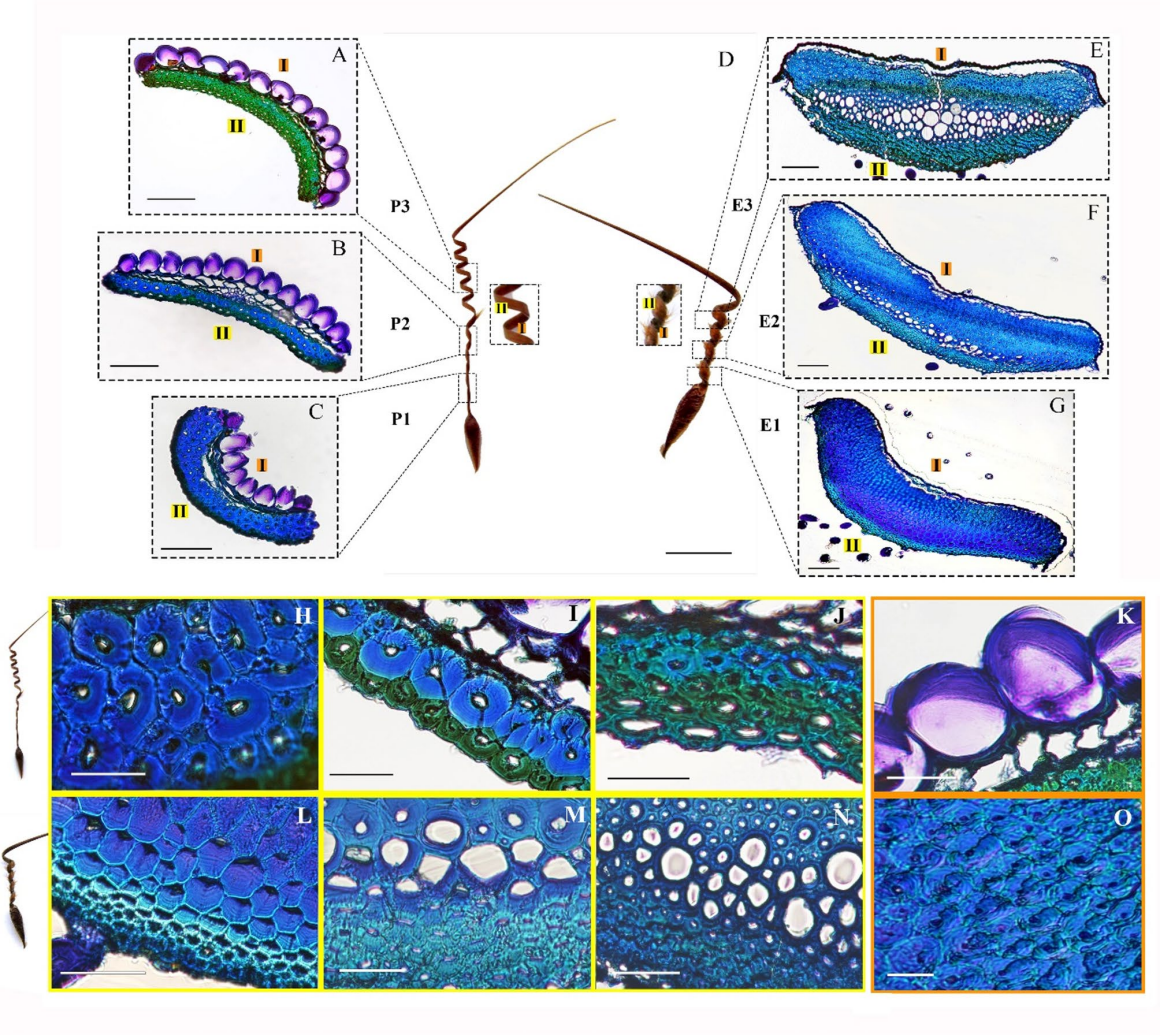
Morphological and histological comparison of *Pelargonium* and *Erodium* along the entire awn structure. The histological sections were stained with Toluidine Blue O. **A**, **B**, and **C** correspond to the awn regions P3, P2, and P1, respectively. D, displays the macroscopic differences between the two awns when placed side by side. **E**, **F**, **G**, correspond to the awn regions, E3, E2, E1, respectively. **H**, **I**, and **J** are detailed images of the outer layer (II) in *Pelargonium* in the regions, P1, P2, and P3. **K**, shows a detailed image of the inner layer (I). **L**, **M**, and N are detailed images of the outer layer (II) in *Erodium* in the regions E1, E2, and E3. **O**, shows a detailed image of the inner layer (I). The labels from image A to C and E to G refer to the inner layer (I) and the outer layer (II). The scalebar is 100 μm for A, B, C, E, F, and G; 5 mm for image D; 50 μm for **L** and **N**; and 25 μm for **H**, **I**, **J**, **K**, **M**, and **O**, (N samples = 8). < insert page break here>.

Here, we investigated the macroscopic divergences between the awns of *Pelargonium appendiculatum* (L.f.) Willd. and *Erodium gruinum* (L.) L’Hér. (Fig. 1D). In *Pelargonium*, the awn is lightweight, elongated, and equipped with hairs in the P2 region, with an initial twisting behavior in the P1 region, followed by a distinguishable coiling region in P3. In *Erodium*, the awn is heavier, robust, with a coiling that increases in diameter from E1 to E3 (Fig. S1). In both cases, the awns have a higher increase in length when wet in the coiling region (P3 and E3), with a two-fold increase for *Pelargonium* and three-fold for *Erodium* (Fig. S1).

### Structural analysis

The structural analysis was conducted using a combination of electron and white-light microscopy. Since our starting material consisted of awns detached from the fruit, we were unable to determine the tissues’ origin within the fruit. As a result, we classified the tissues based on their position relative to the coiling, designating them as the inner and outer coiling layers.

Tentative cell type identification was carried out by integrating Toluidine Blue O staining with SEM-based structural analysis. Toluidine Blue O provided preliminary insight into cell wall composition through differential staining, as described by O’Brien et al. (1964). SEM imaging enabled detailed examination of cell shape, wall thickness, and fracture patterns in both sectioned and hand broken samples. These classifications are intended as functional analogies rather than definitive developmental identities. Accordingly, the terms used were applied based on structural features observed, aiming to provide anatomical justification for cell type categorization without asserting developmental origin^26,27^. In the *Pelargonium* sections, we identified two layers, respectively, an inner and outer layer. The inner layer comprises cells resembling large parenchyma-like cells with asymmetric collenchymatous cell wall thickenings (Fig. 1, K; Fig. S3, D). Here, the term “collenchymatous” is used descriptively to indicate uneven primary wall thickenings resembling those found in collenchyma, without implying that these cells are true collenchyma or contribute directly to mechanical support^28^. These cells are present along the entire awn and are responsible for coiling due to the orientation of the cellulose microfibrils in the cell wall seemingly almost perpendicular to the cell long axis, indicative of high MFA (Fig. S4, C, D)^5^.

In the P1 region, *Pelargonium* outer layer comprises cells resembling sclereid-like cells for their thick lignified secondary cell walls (Fig. 1, C, H; Fig. S3, A), characterized by a low MFA indicated by the cell wall fractures at an acute angle to the cell axis (Fig. S4, A)^29^.

In the P2 region, the outer layer transitions into one row of sclereid-like cells adjacent to one resembling fiber-like cells (Fig. 1, B, I; Fig. S3, B). Furthermore, the section widens (Fig. S2, C) due to an increased number of parenchyma-like cells, from 7 to 12, in the inner layer. In the P3 region, the sclereid-like cells are replaced by fiber-like cells, which are characterized by lignified cell walls (Fig. 1, A, J; Fig. S3, C) that fracture at an acute angle to the cell’s axis (Fig. S4, B). Furthermore, there is no variation in width of the sections in this region (Fig. S2, C).

In the *Erodium* sections, we identified three layers, respectively, an inner, connective, and outer layer. The inner layer has cells resembling sclereid-like cells characterized by thick lignified secondary cell walls in all regions and by a symmetric cell wall deposition (Fig. 1, O; Fig. S3, H). These cells are responsible for driving the coiling motion due to the perpendicular orientation of the cellulose microfibrils to the cell’s axis, confirmed by the cell wall fractures (Fig. S4, F, G)^5^. In the E1 region, the connective layer is composed by cells resembling sclereid-like cells, although with thick asymmetric cell wall deposition, while the outer layer has densely packed cells fiber-like cells characterized by lignified cell walls (Fig. 1, G, L; Fig. S3, E). The sclereid-like cells in the connective layer are characterized by a low MFA, as shown by the acute cell wall fracture to the cell’s axis (Fig. S4, E). In the E2 region, the connective layer contains either thin-walled cells or the remaining sclereid-like cells with thick asymmetric cell walls (Fig. 1F, M; Fig. S3, F, G). Furthermore, we observed an increased in thickness of the outer layer composed by the densely packed fiber-like cells accompanied by a decrease in width of the entire section (Fig. S2, C). In the E3 regions, the connective and outer layer remain similar in cell type composition, i.e. thin-walled and fiber-like cells, respectively (Fig. 1E, N; Fig.S3, G). Although an increase in thickness of the connective layer was observed, together with a decrease in section width (Fig. S2, C).

### Nanoindentation test

A nanoindentation test assessed externally the inner (I) and outer (II) layer stiffness of the two species’ awns in the three regions, respectively P1, P2, P3 for *Pelargonium* and E1, E2, E3 for *Erodium*. In *Pelargonium* stiffness decreased in the outer layer from region P1 to P3, while in *Erodium* it increased from region E1 to E3 (Table 1). The differences in stiffness between the regions were statistically significant in both species (*p* < 0.01 for *Pelargonium* and *P* < 0.001 for *Erodium*, One-way ANOVA test), suggesting substantial differences in the outer tissue layers between the regions of *Pelargonium* and *Erodium*, despite the small sample size (*N* = 5).The Young’s modulus measurements for the inner layer in *Pelargonium* showed a higher stiffness values for P1 region compared to P2 and P3, whereas in *Erodium* we observed a different trend with a stiffness increase in E2, compared to E1 and E3 (Table 1). The difference in stiffness between the regions, both in *Pelargonium* and *Erodium*, were statistically significant (*p* < 0.01, One-way ANOVA test), indicating substantial variation between the regions despite the limited sample size (*N* = 5). Overall, these results indicate that *Pelargonium* and *Erodium* awns exhibit opposite stiffness trends, in particular: *Pelargonium* shows a decrease in stiffness from P1 to P3; while *Erodium* shows an increase from E1 to E3.

**Table 1:** The table reports the stiffness values of the inner (I) and outer (II) layers from regions P1, P2, P3, and E1, E2, and E3 (*N* = 5).

Young’s modulus [GPa]	1		2		3	
	I	II	I	II	I	II
*Pelargonium*	1.10 ± 0.33	2.15 ± 0.61	0.63 ± 0.18	1.42 ± 0.28	0.55 ± 0.15	0.94 ± 0.38
*Erodium*	0.73 ± 0.23	0.34 ± 0.04	3.20 ± 0.68	1.36 ± 0.22	1.73 ± 0.74	4.17 ± 1.20

### Polysaccharides distribution analysis

We analyzed the internal composition of *Pelargonium* and *Erodium* awns to investigate the distribution of non-crystalline components of the cell wall, responsible for humidity absorption, in contrast to cellulosic crystalline microfibrils which set the directionality of the hygroscopic contraction due to their hydrophobic nature^30,31^. White light, epi-fluorescence microscopy, and Raman spectroscopy were used for this analysis.

**Fig. 2:**
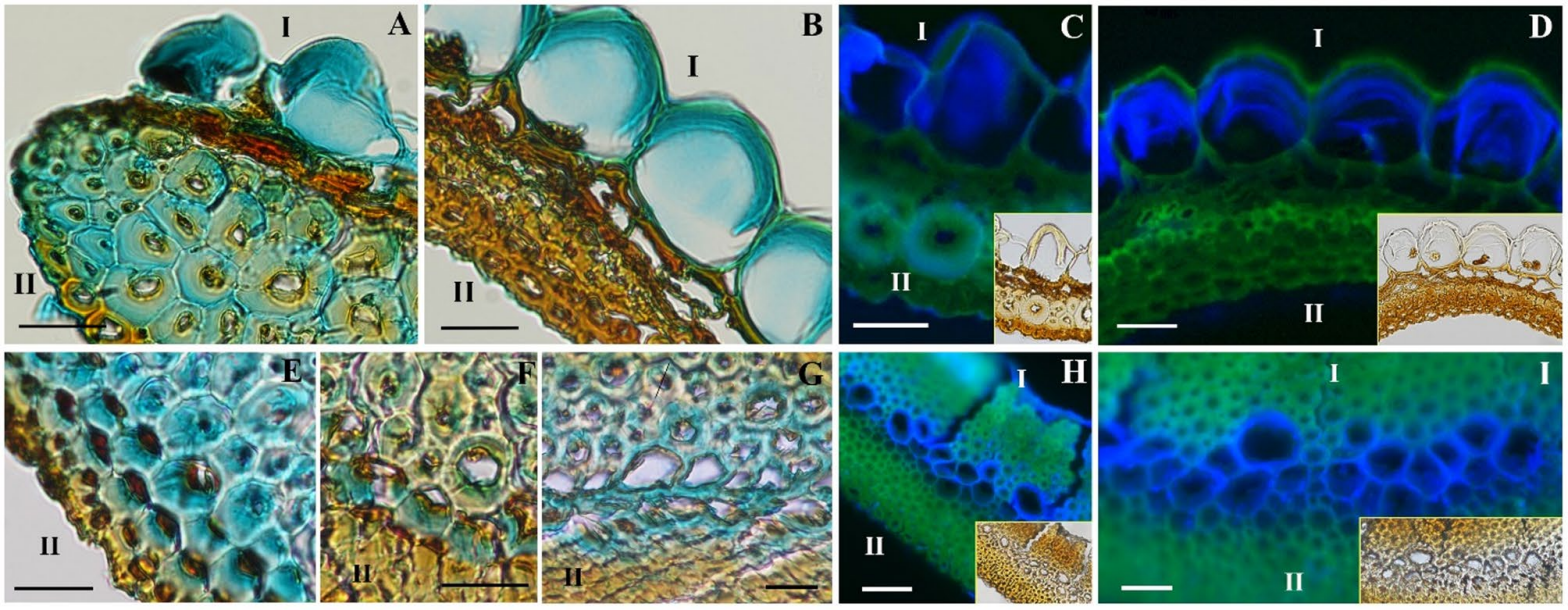
Composition analysis using the dyes Alcian blue and Calcofluor white. **A** and **B** are *Pelargonium* sections from the P1 and P3 region, stained with Alcian blue. **C** and **D** are *Pelargonium* sections from the P1 and P3 region, stained with Calcofluor white. **E**, **F**, and **G** are *Erodium* sections from E1, E2, and E3 region stained with Alcian blue. **H**, **I**, are *Erodium* sections from E3 region stained with Calcofluor white. The labels from image A to I refer to the inner layer (I) and the outer layer (II). The scale bar is 50 μm for H and 25 μm for A, B, C, D, E, F, G, and I (N samples = 5).

In the *Pelargonium* P1 region, pectin/hemicellulose localization with the Alcian blue stain was detected on the external border of sclereid-like cells and in the asymmetric cell wall of the parenchyma-like cells (Fig. 2, A), which were stained across all three regions (Fig. 2, B). No staining was present in the fiber-like cells forming the outer layer in region P3. Calcofluor white staining for cellulose showed similar localization: in region P1, a slight fluorescence was visible on the outside border of the sclereid-like cells and cell walls of the parenchyma-like cells (Fig. 2, C). The latter were similarly stained across all three regions (Fig. 2, D).

In *Erodium*, instead, the histochemical stains only colored the connective layer. In the E1 and E2 regions, pectin/hemicellulose were localized in the sclereid-like cells of the connective layer (Fig. 2, E, F) and in region E3 in the thin-walled cells (Fig. 2, G). Cellulose detection was slightly different: in the E1 and E2 regions, no fluorescence was visible, contrary to the E3 region, where its fluorescence was visible in the thin-walled cells (Fig. 2, H, I). The absence of Calcofluor white fluorescence in different cell types of both species could be attributed to the presence of lignin, which can inhibit the binding of the stain^32^as observed in sclereid-like cell visible in Fig. 2C.

**Fig. 3:**
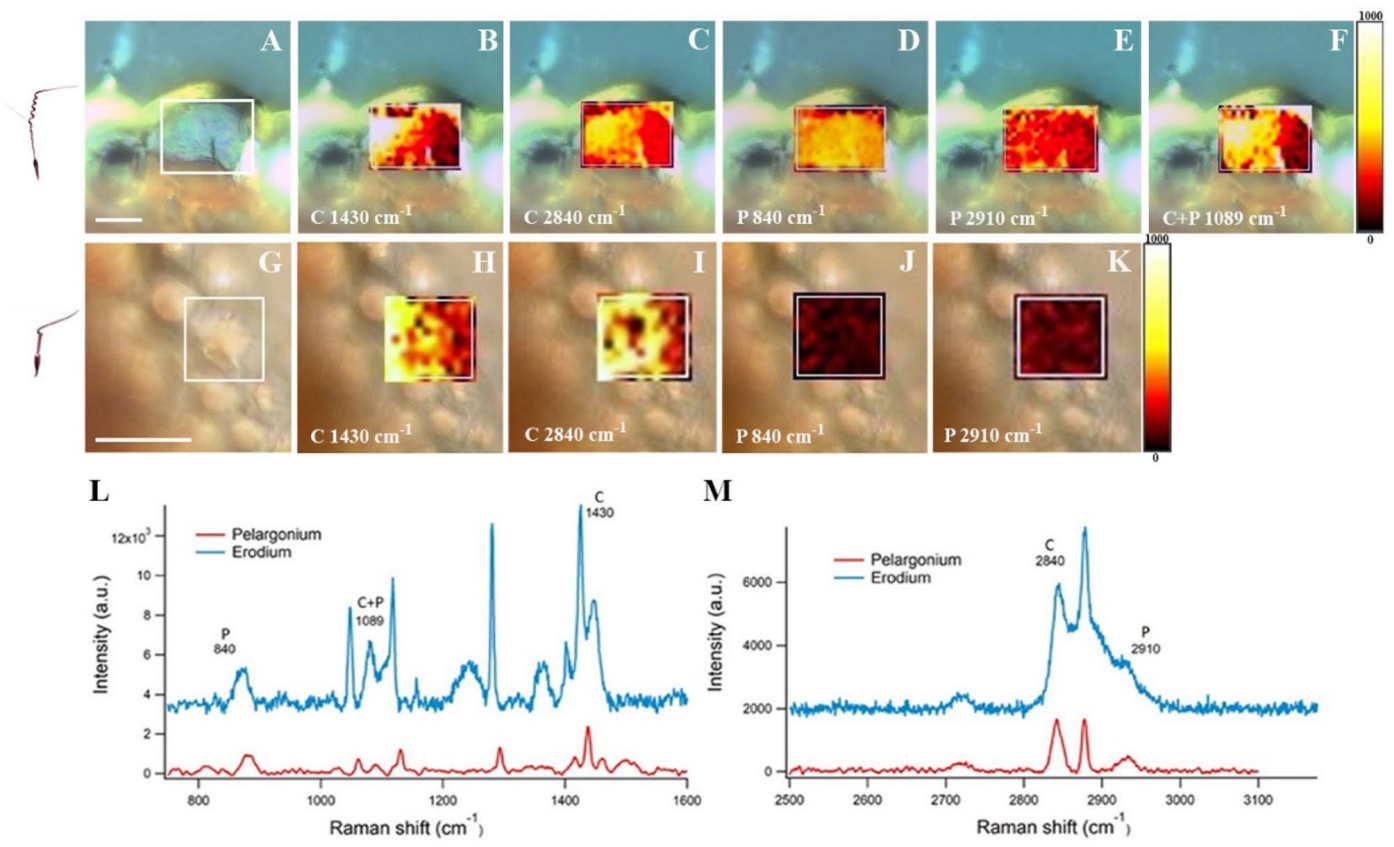
Composition analysis using Raman spectroscopy. **A**, is the parenchyma like cell analyzed in *Pelargonium*. **B**, corresponds to the distribution of the cellulose peak at 1430 cm^−1^ assigned to in-plane CH bending. **C**, corresponds to the cellulose -CH2 stretching band distribution at 2840 cm^−1^. **D**, corresponds to pectin (COC) skeletal mode distribution at 840 cm^−1^. **E**, corresponds to the pectin -CH2 stretching band distribution at 2910 cm^−1^.**F**, corresponds to the distribution of cellulose and pectin shared peak at 1089 cm^−1^ assigned to the asymmetric in-plane ring stretch. **G**, is the connective layer cell analyzed in *Erodium*. **H**, corresponds to the distribution of the cellulose peak at 1430 cm^−1^ assigned to in-plane CH bending. **I**, corresponds to the cellulose -CH_2_ stretching band distribution at 2840 cm^−1^. **J**, corresponds to pectin (COC) skeletal mode distribution at 840 cm^−1^. **K**, corresponds to the pectin -CH_2_ stretching band distribution at 2910 cm^−1^. Raman spectra with the wavelengths of the bands detected for *Pelargonium* and *Erodium*. **L**, Raman spectra of *Pelargonium* and *Erodium* between 800 to 1600 cm^−1^ wavelengths. **M**, Raman spectra of *Pelargonium* and *Erodium* between 2500 to 3100 cm^−1^ wavelengths. The scale bar is 20 μm for A and 50 μm for G, (N samples = 3).

To further confirm the observed polysaccharide composition, Raman spectroscopy analysis was conducted, focusing on the coiling regions of the awns, specifically P3 and E3. Pectin and cellulose were distinguished, although differentiation between cellulose and hemicellulose was not possible due to their similar peaks. In *Pelargonium*’s parenchyma-like cells, cellulose and pectin bands were detected with wavelengths, respectively, 2840 and 1430 cm^−1^ for cellulose (Fig. 3, B, C), 2910 and 840 cm^−1^ for pectin (Fig. 3, D, E), and a 1089 cm^−1^ ordinary band for pectin and cellulose (Fig. 3, F). In *Erodium*’s connective layer cells, cellulose was detected with wavelengths, respectively, at 2840 and 1430 cm^−1^ (Fig. 3H, I), while pectin was present in small amounts with 2910 and 840 cm^−1^ wavelengths (Fig. 3, J, K), confirming the histochemical results.

### Structure modification analysis

To investigate the sensitivity of the structures to relative humidity, we observed the behavior of cut and hand-broken sections of the awns in the coiling region using E-SEM. In *Pelargonium* cut sections (Movie S1), the large parenchymatic-like cells had the highest visible expansion compared to the fiber-like cells. Furthermore, on hand-broken sections (Fig. S5A, B), aqueous microbubbles formed on the cell wall surfaces, implying water attraction. Similarly to humidity, the addition of water to the sections observed under white light microscopy, caused significant expansion, particularly in the parenchyma-like cells (Movie S2).

For *Erodium* sections, instead, it was difficult to differentiate between a differential expansion of the cells when exposed to increasing humidity (Movie S3). Moreover, in hand-broken sections (Fig. S5C, D), we observed an expansion of the inner coiling cells compared to dry conditions, but no aqueous microbubbles were visible, as in *Pelargonium*. However, by observing the sections with white light microscopy, the addition of water caused a high expansion of the cells in the connective layer, leading to cell closure, not visible by increasing the humidity alone (Movie S4). This indicates a different sensitivity to humidity and water presence. Additionally, in *Erodium*, the duration of cell expansion was double the time compared to *Pelargonium* cells, as shown by the speed of the movies (Movie S2 and S4).

### Biomechanical and aerodynamic analysis

The diversification in structure and composition within the Geraniaceae family may be attributed to their varying mechanisms. In the following section, the biomechanical and aerodynamic properties of the two species are examined, with fruit detachment measurements focusing exclusively on the mechanical parameters of the awn involved in the process.

**Fig. 4:**
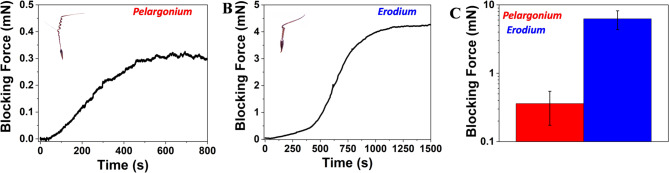
Biomechanical analysis of *Pelargonium* and *Erodium* awns. The deformation of the awn was measured from wet to dry conditions. **A**, the evolution of the blocking force of *Pelargonium*. **B**, the evolution of the blocking force of *Erodium*. **C**, comparison of the blocking forces of *Pelargonium* and *Erodium* at maximum values (N samples = 3).

The ability of Geraniaceae seeds to be explosively launched from the fruit is mediated by the elastic energy of the uncoiled awns, stored during fruit drying and abruptly transformed into kinetic energy causing the ballistic launch. To evaluate the elastic energy stored in tissue during the ballistic launch, we measured the blocking force at the tip of the lever as a function of the variation of relative humidity. Blocking forces (Fig. 4A-C) were measured with the setup reported in Figure S6A, B from wet to dry conditions. The blocking forces were 0.36 ± 0.17 mN and 6.27 ± 1.92 mN, respectively, for *Pelargonium* and *Erodium*. The difference in force between the two groups was statistically significant (Welch’s t-test, *p* < 0.05), indicating a substantial effect despite the limited sample size (*N* = 3). From the blocking forces, we estimated the elastic energy of the two species considering the model provided by Evangelista et al., 2011^15^, where *Pelargonium* resulted U_el_ ≈ 5.6 µJ and *Erodium* U_el_ ≈ 20.1 µJ (see section S1 for formula). The higher blocking force and elastic energy of *Erodium* compared to *Pelargonium* confirm the greater inclination of *Erodium* in using the ballistic launch for aerial dispersion.

To analyze the soil exploration and self-burial ability of the awn we measured its dynamic and kinematic deformation under different relative humidity conditions^13,15^.

**Fig. 5:**
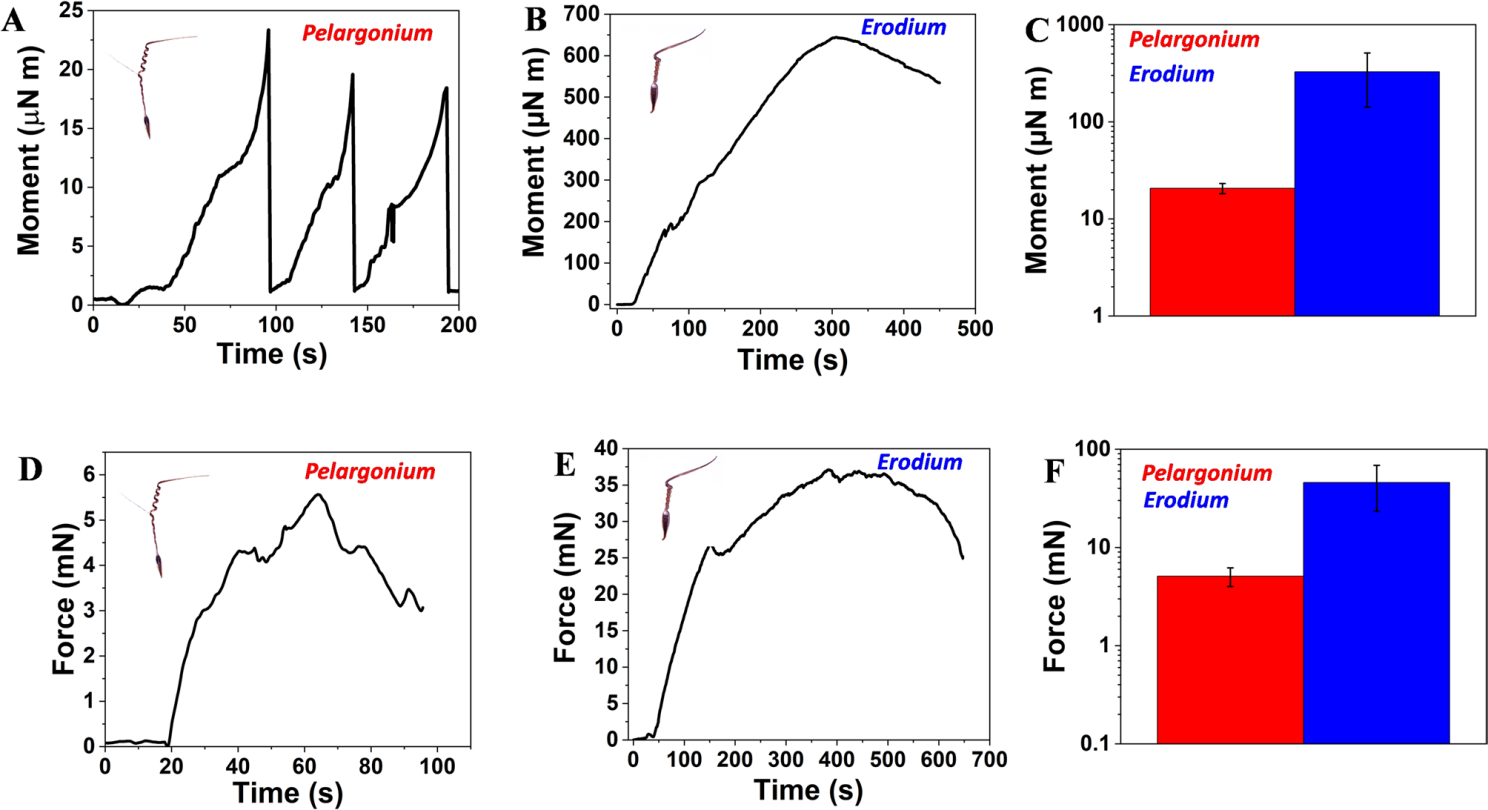
Biomechanical analysis of *Pelargonium* and *Erodium* awns. **A**, the evolution of the moment in *Pelargonium*. **B**, the evolution of the moment in *Erodium*. **C**, comparison of the moment for *Pelargonium* and *Erodium* at equilibrium values. **D**, the evolution of the extensional force in *Pelargonium*. **E**, the evolution of the extensional force in *Erodium*. **F**, comparison of the extensional forces for *Pelargonium* and *Erodium* at the equilibrium values (N samples = 3). (N samples = 3). For A, C, D and F, therefore only for *Pelargonium*, the data is reproduced and adapted with permission. (Cecchini, L.; Mariani, S.; Ronzan, M.; Mondini, A.; Pugno, N. M.; Mazzolai, B. 4D Printing of Humidity-Driven Seed Inspired Soft Robots. *Adv. Sci.* 2023, 2205146). Copyright 2023, John Wiley and Sons (CC BY 4.0 DEED).

For the dynamic measurements of the awn, moment (Fig. 5A-C) and extensional forces (Fig. 5D-F) were measured with the setup reported in Figure S6C, D from wet to dry conditions and compared to the theoretical results obtained using a model developed by Ha et al., 2020 ^13^ in which the hygroscopic structure is separated into a trilayer (see section S2 for formula). The model was adapted using experimental mechanical parameters as reported by Cecchini et al., 2023 ^14^. The extensional forces were 5.1 ± 1.1 mN and 45.9 ± 22.6 mN (5.6 mN and 53.3 mN for the model), while the moments were 20.7 ± 2.5 and 528 ± 54 µN m (57.7 and 658.5 µN m for the model), respectively for *Pelargonium* and *Erodium.* The difference in force between the two groups was statistically significant (Welch’s t-test, *p* < 0.05), indicating a substantial effect despite the limited sample size (*N* = 3). The difference between the two groups of moments was significant too (Welch’s t-test, *p* < 0.01) (*N* = 3); this indicates a substantial effect size despite the limited sample size. The results obtained were comparable with the model, except for the higher moment value in the *Pelargonium* model explained by the buckling effect during the measurement, i.e., the loss of contact of the awn with the force sensor due to its cylindrical axis deformation. A matching between the model and moment measured in *Erodium* was probably due to the less deformable and stiffer nature of the awn. The dynamic measurement demonstrates that once on the ground, *Erodium* could use high extensional forces and moment for self-burial and soil exploration more than *Pelargonium*.

Kinematic deformations of the awn at varying relative humidity conditions were monitored by angular displacements (θ_30_, °), from which it was possible to estimate the hygroscopic angular sensitivity (°/RH), the response time (Rt), diffusion coefficient (D) and the angular velocity (ω, °/s). Already from a first qualitative evaluation with water aerosols it was clear that the awn of *Pelargonium* was able to respond and deform more rapidly than *Erodium* (Movie S5).

**Fig. 6:**
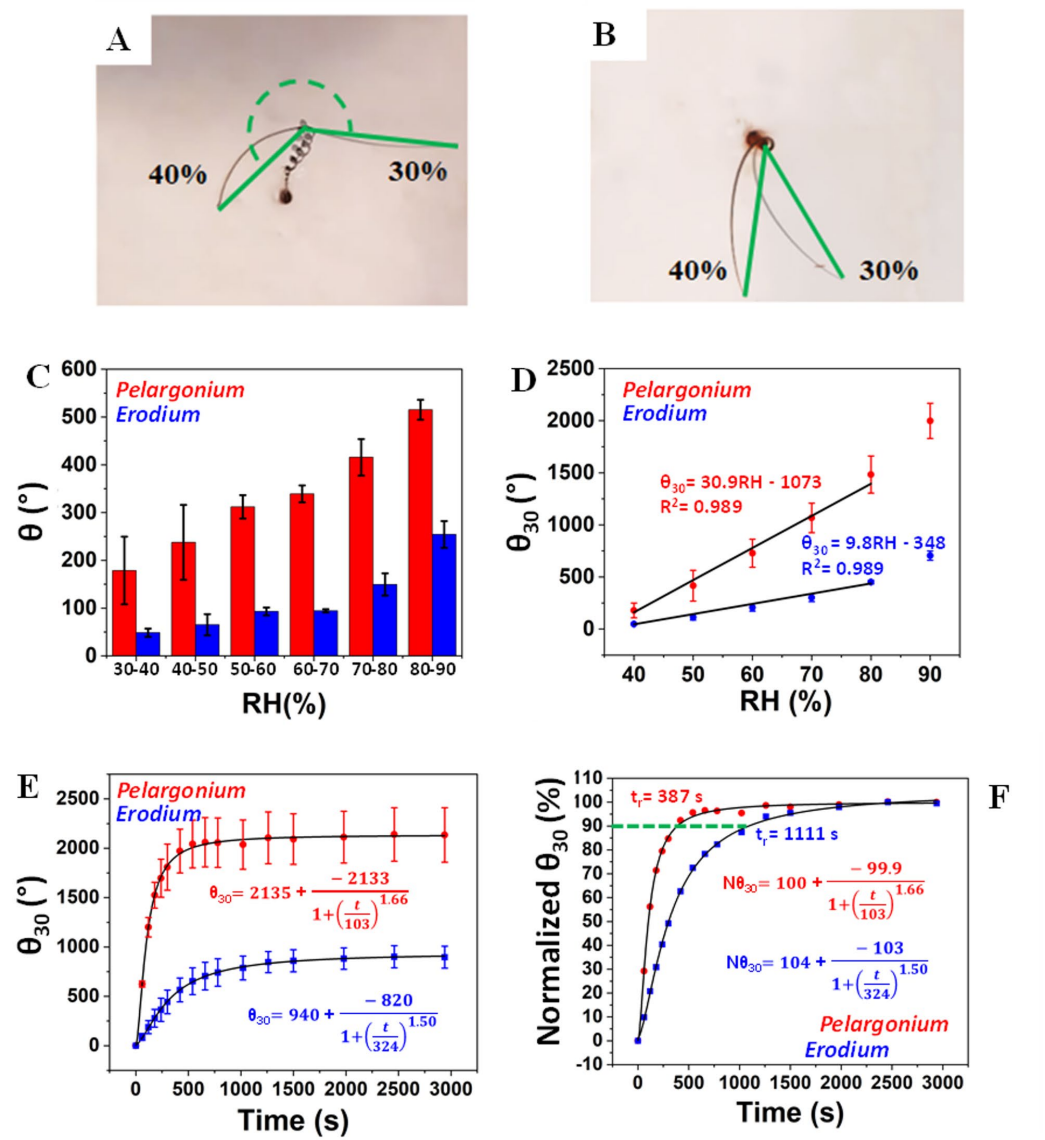
Biomechanical analysis of *Pelargonium* and *Erodium.* **A**, an example of angle measurement of the lever rotation from 30–40% for *Pelargonium*. **B**, an example of angle measurement of the lever rotation from 30–40% for *Erodium*. **C**, diagram showing Angle displacement (θ, °) every 10% step variation over different RH values for *Pelargonium* and *Erodium* lever. **D**, reports angle displacement compared to RH = 30% (θ_30_) over different RH values for *Pelargonium* and *Erodium*. Linear fitting functions are also reported. **E**, reports angle displacement compared to RH = 30% (θ_30_) over time (s) for *Pelargonium* and *Erodium* during a single, stepwise, and abrupt variation of Relative Humidity (%RH) from 30–90%. **F**, reports the same data in E but normalized. Sigmoidal logistic functions for E and F are also reported (N samples = 3).

The angular displacements (θ_30_, °) of *Pelargonium* and *Erodium* awn tail (Fig. 6A, B, Fig. S7A-H for *Pelargonium* and Fig.S7I-P for *Erodium*) were measured as a function of Relative Humidity (RH) compared to the initial value at RH = 30% (θ_30,_ °) at different RH until reaching RH = 90%, with 10% step variations (Table S1A, B, Movie S6 and Movie S7). The values of θ_30_ were sampled when the equilibrium was reached.

The θ values (i.e., angle displacement every 10% step variation over different RH) resulted higher in *Pelargonium* than *Erodium*, with a 3.68-fold at RH = 30–40% and a 2.02-fold at RH = 80–90% (Fig. 6C). Figure 6D reports the θ_30_ (°) over %RH for both awns, fitted as linear functions: θ_30_(°) = 30.9RH(%) – 1073(°) (R² = 0.989) and θ_30_(°) = 9.8RH(%) – 348(°) (R² = 0.989) for *Pelargonium* and *Erodium*, respectively. The slope of the linear fitting shows that *Pelargonium* has a hygroscopic angular sensitivity (°/RH) to humidity variation 3-fold higher than *Erodium*.

Figure 6E, F show the variation of θ_30_ (not normalized and normalized) over time (s) during a single, stepwise, and abrupt variation of Relative Humidity (%RH) from 30 to 90% (Movie S8) for the estimation of the angular velocity (ω, °/s) and of the response time (t_r_, s). The values of θ_30_ were sampled when the equilibrium was reached. The trends were well fitted (R^2^ = 0.999) by sigmoidal/logistic function both for *Pelargonium* and *Erodium*. From the analysis of the fitting curves and from the search of the maximum at the zeroing of the second derivative we obtained a maximum ω equal to 12.6 °/s and 1.54 °/s, respectively after 44.5 and 111 s, for *Pelargonium* and *Erodium*, with *Pelargonium* roughly 8-fold that of *Erodium*.

The response times (t_r_, s) of the two awns estimated from the 90th percentile of the graphs reported in Fig. 6F with normalized angular variation (Nθ_30_, %) were respectively, 387 ± 37 s and 1111 ± 126 s for *Pelargonium* and *Erodium* (N samples = 3). The difference in mean values between the two groups (387 ± 37 s vs. 1111 ± 126 s, *N* = 3) was statistically significant (Welch’s t-test, *p* < 0.01), indicating a robust effect despite the limited sample size. From the response times, we were also able to calculate the diffusion coefficient (D) of water in the inner tissue^14^*D = h*_*c*_^*2*^*/t*_*r*_, where *h*_c_ is the thickness of the inner layer (Length 3, section I, Fig. S2C), and *t*_s_ is diffusion time. Interestingly, we obtained a lower D through the inner layer for *Pelargonium*, 6.7 ± 0.1 × 10^−12^ m^2^/s^−1^, in respect to *Erodium*, 8.4 ± 0.3 × 10^−12^ m^2^/s^−1^. The difference between the two diffusion coefficients was statistically significant (Welch’s t-test, *p* < 0.05), indicating a reliable difference despite the limited sample size (*N* = 3).

The data confirmed that *Erodium* is less sensitive at varying humidity conditions compared to *Pelargonium*, indicating that *Erodium* will probably remain located in the area where it is launched rather than exploring soil, as previously observed^17^. Despite the low sensitivity to varying humidity conditions *Erodium* had a higher water diffusion coefficient of the inner layer, explainable by the presence of hydrophobic material, lignin and its derivates, which can create weak bonds with water allowing faster water dynamics^32^.

In addition, the helical motion of the awn could be predicted considering the radius (R) and the pitch (P) as a function of RH(%)^14^(Fig. S8A). A more significant variation in *Pelargonium* than *Erodium* was observed at different RH values (Fig. S8B, C,S9,S10). All the collected kinematic data proved a higher deformation ability of *Pelargonium* compared to *Erodium*, in agreement with the lower flexural and torsional rigidity (storage modulus data reported in Table 1).

Aerodynamics also plays a pivotal role in seed dispersal in the Geraniaceae family. As reported by Evangelista et al., 2011^15^, wind could act as an additive dispersal force, especially for short-range ballistic launches. Indeed, if the drag reduces terminal velocity to a value comparable with wind speeds, wind dispersal is a possibility^15^.

Terminal velocities (v_t_) were measured after dropping the mericarps (Movie S9). The projected surface (A) of the mericarps was also estimated.

Drag coefficients (C_D_) were calculated following Yoo et al.^35^

$$\:{C}_{D}=\frac{2m\times\:g}{{\rho\:}_{air}\times\:{\left({v}_{t}\right)}^{2}\times\:A}$$

where *m* is the mass, *v*_*t*_ is the terminal velocity speed, A is the projected surface, *g* is the gravitational acceleration (9.81 m/s^2^), *ρ*_*air*_ is the density of the air (1.204 kg/m^3^). The data are summarized in Fig. S11.

The resulting C_D_ were 1.78 ± 1.41 and 2.48 ± 0.98, respectively, for *Erodium* and *Pelargonium*.

The difference between the two groups was not statistically significant (Welch’s t-test, *p* < 0.05), indicating no clear evidence of difference with the current sample size.

Although the data is dispersed due to error propagation with relative standard deviations (RSD%) at around 50% (in line with previous reports^15^), they demonstrate the superior aerodynamic performance of *Pelargonium* with a tendency towards drag and wind dispersion. This was also intuitively shown by the different *v*_*t*_, i.e., 1.95 ± 0.14 and 3.65 ± 0.91 m/s for *Pelargonium* and *Erodium*. The difference between the two diffusion coefficients was statistically significant (Welch’s t-test, *p* < 0.05), indicating a reliable difference despite the limited sample size (*N* = 3). This data further confirms that in *Erodium* the maximum distances from the fruit are reached through a ballistic launch.

## Discussion

### Morphological and structural analysis

*Pelargonium* has a lightweight, elongated, hairy awn with an internal bilayer structure, characterized by an initial twisting followed by a coiling of the awn (Fig. 1D; Fig. S1A). We observed the replacement of sclereid-like cells with fiber-like cells in the outer layer along the awn (Fig. 1, H-J). Fibers and sclereid-like cells are sclerenchyma cells, which, generally enhance the mechanical functions of structures, such as resisting strains, bending, weight, and pressure^11,36^. We measured an increase in stiffness in the outer layer in the presence of sclereid-like cells in the P1 region (Table 1). On the contrary, in the same outer layer, we measured a reduction in stiffness of the awn in the presence of the fiber-like cells in the P3 region (Table 1). Given that the inner layer overall remains unchanged, the presence of the fiber-like cells contributes to the awn’s flexibility, therefore coiling. In line with this hypothesis, it was observed that fiber-like cells provide high tensile strength to structures^37^ which explains the high extension of the coiling region we observed compared to the base when wet (Fig. S1B). *Erodium* has a heavyweight, robust and rigid morphology explained by the simultaneous presence of sclerenchyma cells, i.e., fibers and sclereid-like cells, which together contribute to the mechanical properties of the awn (Fig. 1, L-O; Fig. S1A). The awn has an increased coiling diameter from the base to the coiling region (Fig. 1E-G), previously justified by gradual changes in cellulose microfibril structure^9^. We observed an increase in thickness of the outer and connective layers, formed by fiber-like and thin-walled cells, respectively, and a decrease in width of the awn section from the base to the coiling region (Fig.S2C), implying the contribution also of these factors to coiling of the awn. In line with this hypothesis, the connective layer was already proposed to favor the outer layer’s tendency to increase the coil radius, instead of resisting it^5^. Contrary to *Pelargonium*, we observed an increase stiffness of the outer layer in the coiling region, which can be attributed to the increased thickness of the connective layer (Table 1), as well as the cell wall composition, which contains high concentrations of ferulic acid, as previously observed^34^. However, despite the high stiffness in the coiling region, we observed a high extension of the awn when wet compared to the base region (Fig. S1B). This condition can be explained by the increase in presence of the fiber-like cells in the coiling region (Fig.S2C), which can provide high tensile strength^36^.

### Composition and structure modification analysis

In *Pelargonium* the inner layer cells (Fig. 1, K), responsible for the coiling motion, have a particular asymmetric cell wall thickening, which we previously called “collenchymatic thickening” due to its similarity to collenchyma cells for the uneven cell wall deposition and composition, i.e. polysaccharides, specifically cellulose, pectin and hemicellulose (Figs. 2A-F and 3A-D)^28^. We expect that water absorption occurs thanks to polysaccharides in the non-crystalline matrix, and not by cellulose microfibrils which represent the stiff component of the cell wall^38^. A study on the deformation of cellulose microfibrils under different humidity conditions showed that non-crystalline structures were capable of re-arranging on the surface of the microfibrils, making them moisture accessible^39^. These observations could explain the aqueous microbubble formation we observed on the transversal surface of cellulose microfibrils at high humidity conditions, representing, perhaps, potential binding sites (Fig. S5C). Furthermore, the transversal expansion of the inner layer cell walls became evident when exposed to increased humidity and water. This flexible behavior indicates the presence of amorphous cellulose (Movie S1; Movie S2), which can influence the transversal properties of the cell walls^40,41^. Overall, the results from the composition and structure modification analysis demonstrate a high sensitivity of *Pelargonium* to high relative humidity conditions.

In *Erodium* the inner layer, responsible for the coiling, consists of sclereid-like cells with thick symmetric cell walls (Fig. 1, O). These sclereid-like cells were not stained with the histochemical dyes for polysaccharides implying the presence of lignin affecting polysaccharides detection with dyes. Therefore, we expect these walls to be composed of a mixture of hydrophobic compounds, such as lignin, and its derivates and crystalline cellulose microfibrils, which form weak binding sites with water. These observations are confirmed by the exposure of hand-broken awns and cut sections to increasing humidity conditions. We observed an overall expansion of the cells with no formation of aqueous microbubbles on the surface of the cells, i.e., of visible binding sites (Fig. S5D), implying a lower sensitivity to humidity compared to *Pelargonium*. Pectin and hemicellulose were identified in the connective layer of the E1 and E2 regions, with the latter showing an expansion of sclereid-like cells in response to water (Movie S4). Despite some similarities with *Pelargonium’s* inner layer cells, these cells could function as a connecting tissue between the rigid sclereid-like cells of the inner and fiber-like cells of the outer layer, rather than being humidity responsive.

### Biochemical and aerodynamic analysis

The flexible structure and high humidity response of *Pelargonium*’s awn influenced the biomechanical and aerodynamic analysis (Movie S5, Movie S9). Low blocking forces and elastic energy obtained demonstrate that the detachment of the mericarp carrying awn from the fruit is less likely to occur through an explosive launch (Fig. 4, A, C), and can be justified by the presence of by fiber-like cells which reduce stiffness (Table 1)^15^. Whereas, our aerodynamic analysis demonstrated the superior aerodynamic performance of *Pelargonium* with a tendency towards drag and wind dispersion, due to the awn’s lightweight and the presence of parachute-like hairs, which enhance the drag, as previously suggested^5^. *Pelargonium* was less efficient in actuating self-burial and soil exploration due to the low moment and extensional forces in time exerted by the awn at high humidity conditions, both in the experimental results and in the laminate composite model (Fig. 5, A, D). However, the high angular displacement of the lever that we observed at different relative humidities (Fig. 6, C, D), indicates an on-soil dispersion driven by substantial humidity variation. Despite the high sensitivity to varying humidity conditions, also confirmed by the low response time and high angular velocity, *Pelargonium* exhibited a low water diffusion coefficient. This is explainable by the presence of hydrophilic compounds in the parenchyma-like cell walls which form strong hydrogen bonds with water, thereby slowing its diffusion^4^.

The structural and mechanical properties of *Erodium* strongly influenced the biomechanical and aerodynamical analysis (Movie S5). Our results indicate that the detachment of the mericarp carrying awn from the fruit is most likely to occur through an explosive launch due to the high elastic energy (Fig. 4, B, C). This type of detachment has been proposed to occur in stiff materials under high desiccation conditions^15^ a scenario applicable to *Erodium* due to its stiff nature, particularly in the coiling region, as previously described. The use of the explosive launch for the flight from the fruit was confirmed by the low aerodynamic performance, contrary to the high blocking force and estimated elastic energy, indicating that the maximum distance reached away from the fruit occurs through a ballistic launch in *Erodium*. *Erodium* can use high moment and extensional forces for self-burial and soil exploration (Fig. 5, B, E). These results are in agreement with the laminate composite model, especially for extensional forces and observations reported in literature^13,16^. Conversely, it is less sensitive at varying humidity conditions than *Pelargonium* (Fig. 6, C, D), implying that *Erodium* will probably remain in the area where it was explosively launched rather than exploring soil, as previously observed^17^. Despite the low sensitivity to varying humidity conditions, which were noticeable also with the high response time and low angular velocity, *Erodium* resulted in a higher water diffusion coefficient of the inner layer, due to the presence of hydrophobic material, lignin and its derivates, which create weak bonds with water, allowing faster water dynamics^32^.

##  Conclusion

**Fig. 7:**
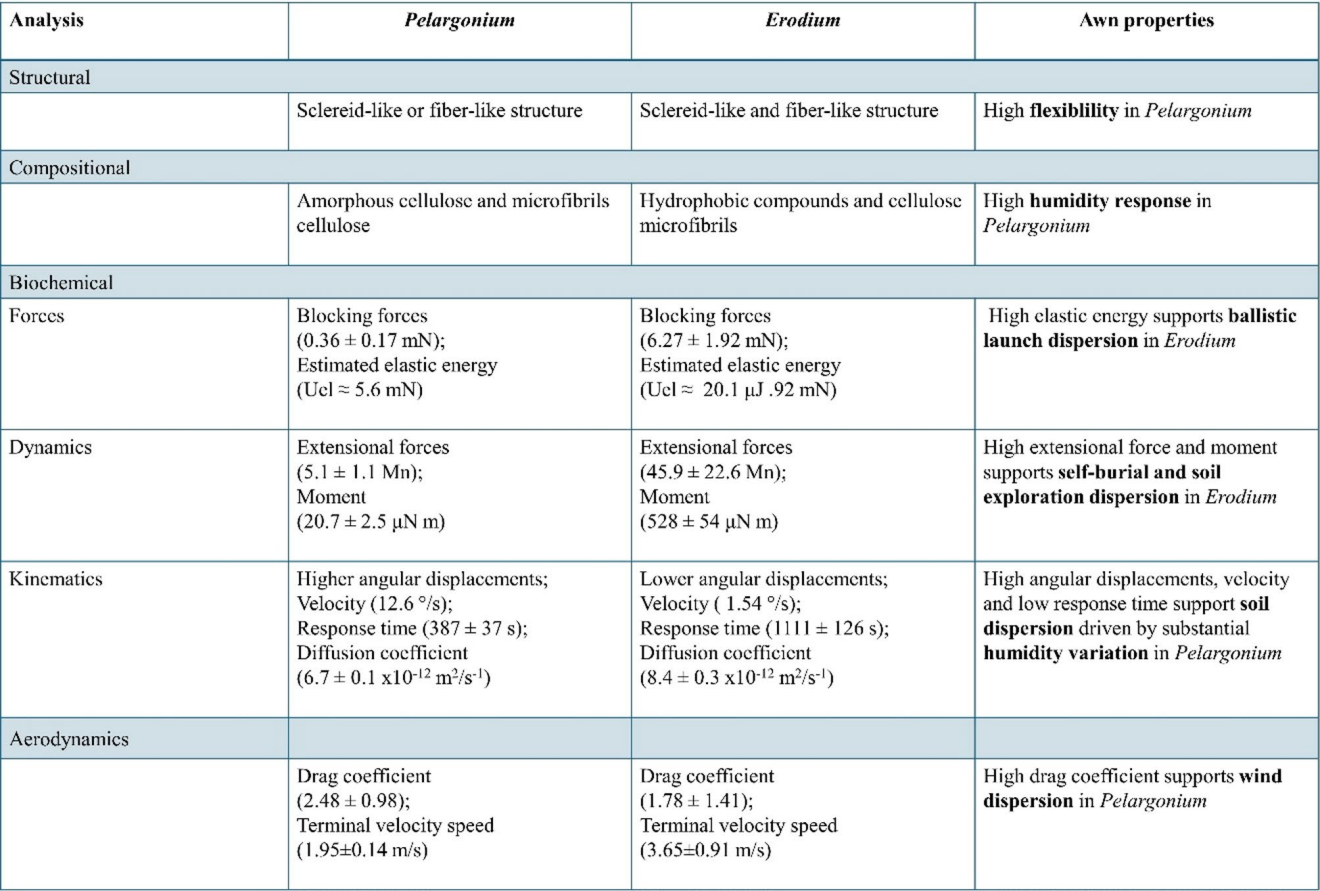
The image reports the main results for each analysis and the resulting awn properties.

In conclusion, both species conserve the trait of being composed of sclerenchyma cells within the structure, though their presence and distribution differ, affecting the awn’s mechanical properties. In addition, the localization and role of polysaccharides in the non-crystalline matrix influence the awn’s water response differently. Our biomechanical and aerodynamic analysis demonstrates that for *Erodium* the high estimated elastic energy facilitates aerial dispersion through ballistic launch followed by soil exploration and self-burial, driven by significant extensional force and moment. In contrast, for *Pelargonium*, a higher drag coefficient supports wind dispersion followed by soil exploration, owing to the large angular displacement under varying humidity conditions, as illustrated in Fig. 7.

These contrasting dispersal mechanisms may reflect evolutionary responses to the distinct climatic and ecological conditions in which *Erodium* and *Pelargonium* species evolved. *Erodium gruinum* (L.) L’Her., native to Iran and commonly found across Mediterranean drylands^42^ experiences relatively low wind speeds^43^ (3–6 m/s, comparable to the terminal velocity of the awn) and limited rainfall^44^ typical of arid and patchy habitats. In such environments, where wind dispersal is less reliable, the evolution of a heavier, spring-loaded awn capable of ballistic seed launch and self-burial could represent an adaptive strategy to enhance local seed deposition and establishment. In contrast, *Pelargonium appendiculatum* (L.f.) Willd., which is native to the southwestern region of South Africa^45^ inhabits a much windier environment, with mean wind speeds reaching approximately 10 m/s^44^, around five times the terminal velocity of its awn. This high wind exposure may have favored the evolution of lightweight, aerodynamic structures optimized for wind-assisted dispersal, coupled with humidity-driven movement for soil exploration. However, this hypothesis requires further assessment and evaluation to verify its validity. We believe that our comparative analysis has clarified the mechanistic aspects of awn dispersion, offering new insights into their movement, driven solely by their structure and biomechanics. Furthermore, we propose a methodology for studying the awn, which can be applied not only to species within the Geraniaceae family but also to other species that use awns as dispersal units. Additionally, this approach could serve as a reference for developing soft robots inspired by Geraniaceae awns.

## Electronic supplementary material

Below is the link to the electronic supplementary material.

Supplementary Material 1

Supplementary Material 2

Supplementary Material 3

Supplementary Material 4

Supplementary Material 5

Supplementary Material 6

Supplementary Material 7

Supplementary Material 8

Supplementary Material 9

Supplementary Material 10

## Data Availability

All data needed to support the conclusions of this manuscript are included in the main text and supplementary information.
