# Supplementary material for: Structural and mechanical properties of humidity-responsive Geraniaceae awns
Source: Sci Rep. 2025 Jul 1;15:21289. doi: 10.1038/s41598-025-09186-6 (PMC12219170; doi:10.1038/s41598-025-09186-6)
Supplement: Supplementary file 10 — Supplementary Material 10 [file 41598_2025_9186_MOESM10_ESM.docx]

Supporting Information

Structural and mechanical properties of humidity-responsive Geraniaceae awns

*Marilena Ronzan^1^*, Stefano Mariani^1^, Luca Cecchini^1,3^, Carlo Filippeschi^1^, Silvia Dante^2^, Nicola Pugno^3,4^, Barbara Mazzolai^1^**

*Marilena Ronzan, Barbara Mazzolai.

Email: marilena.ronzan@iit.it, barbara.mazzolai@iit.it

**This PDF file includes:**

- **Fig. S1**. Morphological characterization of *Pelargonium* and *Erodium* awns;
- **Fig. S2**. Characterization of *Pelargonium* and *Erodium* awns sections;
- **Fig. S3.** Histological analysis of *Pelargonium* and *Erodium* awns by SEM;
- **Fig. S4.** Analysis of hand-broken sections of *Pelargonium* and *Erodium* by SEM;
- **Fig. S5.** Hand-broken sections of *Pelargonium* and *Erodium* observations with ESEM;
- **Fig. S6.** Load-cell-based setup for blocking force extensional forces and moment measurements from wet to dry conditions;
- **Fig. S7.** Angular displacement (θ, °) of *Pelargonium* and *Erodium* vs RH(%);
- **Fig. S8.** Pitch (P) and Radius (R) for *Pelargonium* and *Erodium* at different RH(%);
- **Fig. S9.** Data and pictures of P and R for *Pelargonium* at different relative humidity conditions;
- **Fig. S10** Data and pictures of P and R for *Erodium* at different relative humidity conditions;
- **Fig. S11.** Aerodynamic performance data of *Pelargonium* and *Erodium*;
- **Fig. S12.** A simplified sketch of the awn decomposed into trilayers;
- **Tables S1.** Data of angular displacements;
- **Section S1.** Elastic energy formula from Blocking Force;
- **Section S2.** Modeling of kinematic and dynamic parameters of *Pelargonium* and *Erodium* awns.

**Other Supplementary Materials for this manuscript include the following:**

- **Movie S1.** *Pelargonium* humidity absorption, ESEM;
- **Movie S2.** *Pelargonium* water absorption, white-light microscopy;
- **Movie S3.** *Erodium* humidity absorption, ESEM;
- **Movie S4.** *Erodium* water absorption, white-light microscopy*;*
- **Movie S5.** Awn deformation in *Pelargonium* and *Erodium*;
- **Movie S6.** *Erodium* angular displacements at different RH with 10% step variations;
- **Movie S7.** *Pelargonium* angular displacements at different RH with 10% step variations;
- **Movie S8.** *Pelargonium* and *Erodium* angular displacement during an abrupt variation of RH from 30-90%;
- **Movie S9.** Aerodynamic test of *Pelargonium* and *Erodium*


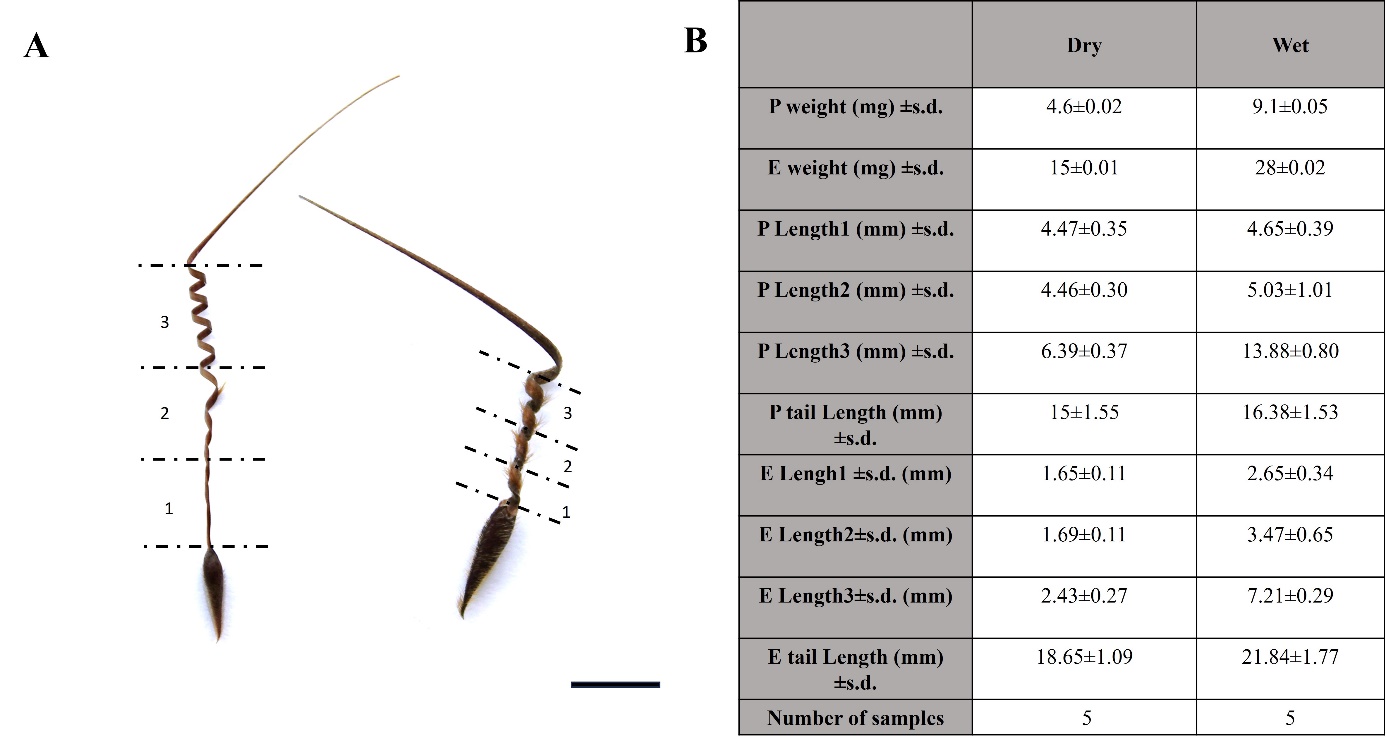


**Fig. S1**. Morphological characterization of *Pelargonium* and *Erodium* awns. A, the awns were divided into three parts, labeled 1, 2, and 3, starting from the region closest to the seed capsule. The scale bar represents 5 mm. B, total dry and wet weights, along with the lengths of the awn segments in both dry and wet conditions.


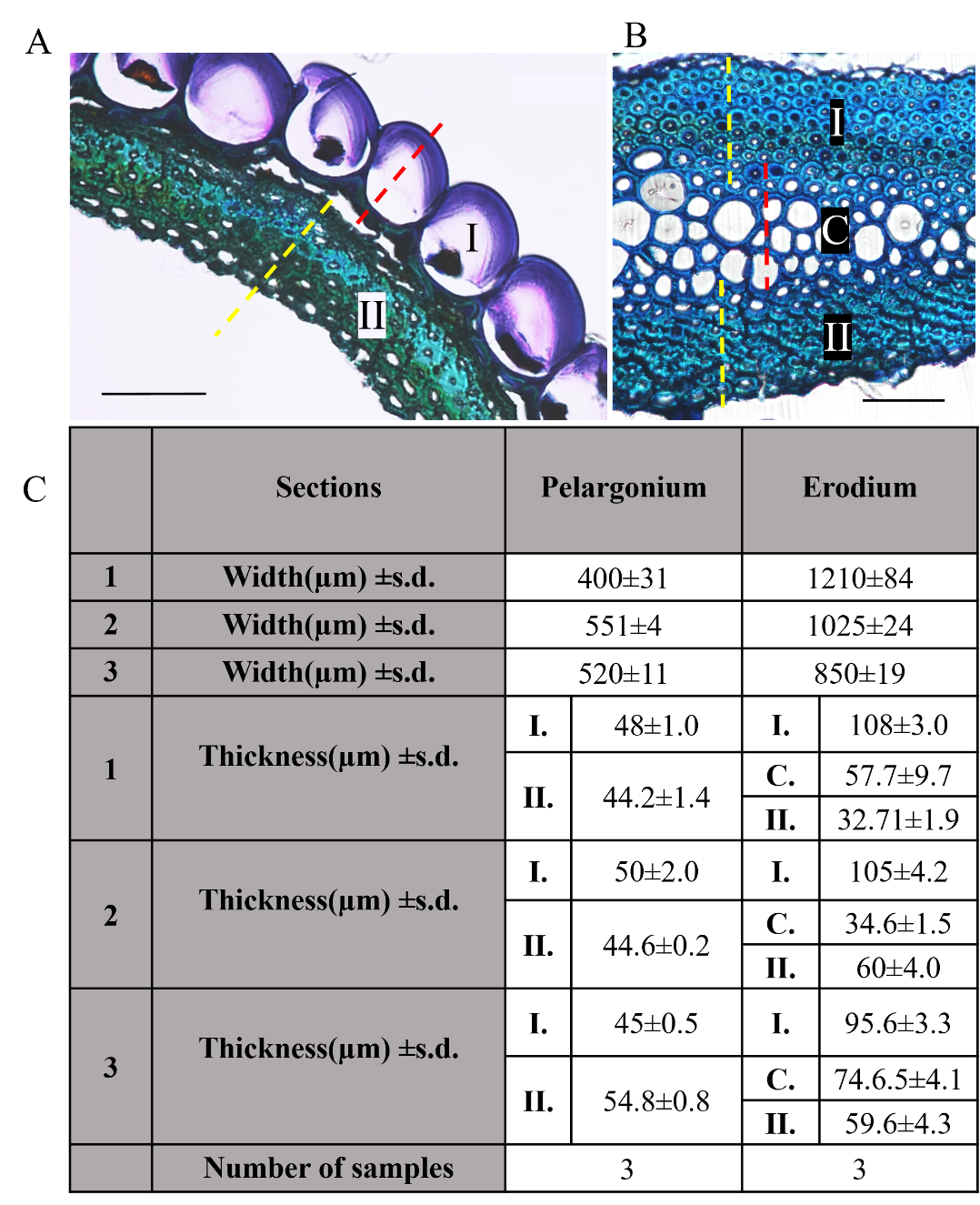


**Fig. S2**. Characterization of *Pelargonium* and *Erodium* awn sections. A, the *Pelargonium* P3 section used for measurements, with a 50 µm scale bar. B, the *Erodium* E3 section used for measurements, with a 50 µm scale bar. C, within each region along the awn (1, 2, 3), histological sections were measured for width and thickness. For the thickness study, the inner (I) and outer (II) layers were measured for *Pelargonium*, while *Erodium* sections were analyzed for the outer (II), connective (C), and inner (I) layers.


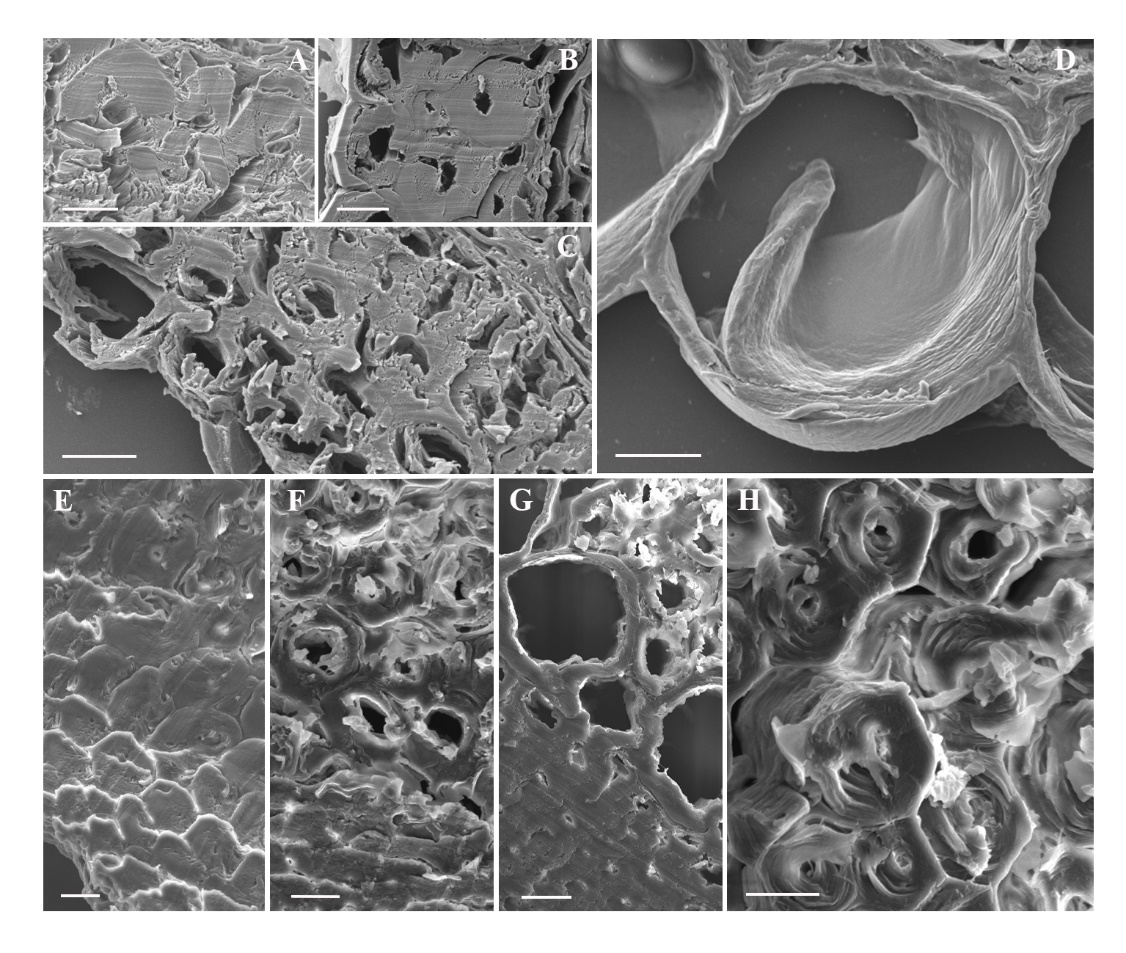


Fig. S3. Histological analysis of *Pelargonium* and *Erodium* awns by SEM. A, B, C, images show the outer layer of the *Pelargonium* awn in the P1, P2, and P3 regions. D, image shows the parenchyma-like cells forming the inner layer of the *Pelargonium* awn. E, F, G, images correspond to the outer and connective layers of the *Erodium* awn in the E1, E2, and E3 regions. H, image shows the sclereid-like cells forming the inner layer in *Erodium*. The scale bar represents 10 µm for all images (A, B, C, D, E, F, G, H; N samples = 3).


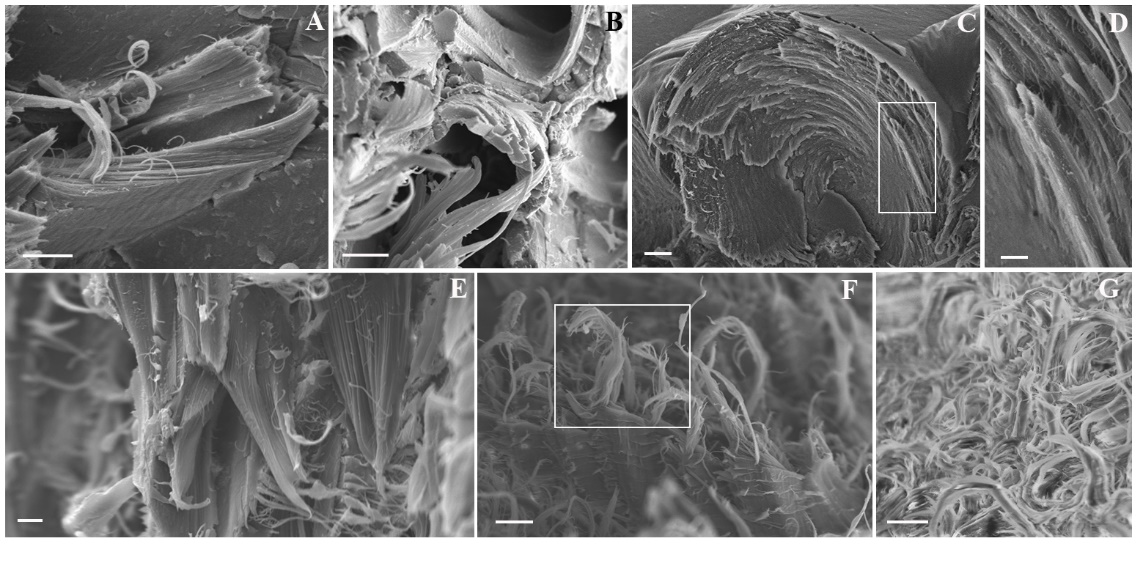


Fig. S4. Analysis of hand-broken awn sections of *Pelargonium* and *Erodium* by SEM. A, shows a broken sclereid-like cell forming the outer layer in *Pelargonium* P1 region. B, shows a broken fibrous-like cell forming the outer layer in *Pelargonium* P3 region. C, shows a broken parenchyma-like cell forming the inner layer in *Pelargonium*. D, provides a detailed view of the microfibrils forming the cell wall of the parenchyma-like cell in C. E, shows broken sclereid-like cells forming the connective layer in *Erodium* E1 and E3 region. F, shows broken sclereid-like cells forming the inner layer in *Erodium*. G, provides a detailed view of the microfibrils forming the sclereid-like cells in F. The scale bar is 3 µm for (A, B, C, D, E) and 10 µm for (F, G) (N samples= 3).


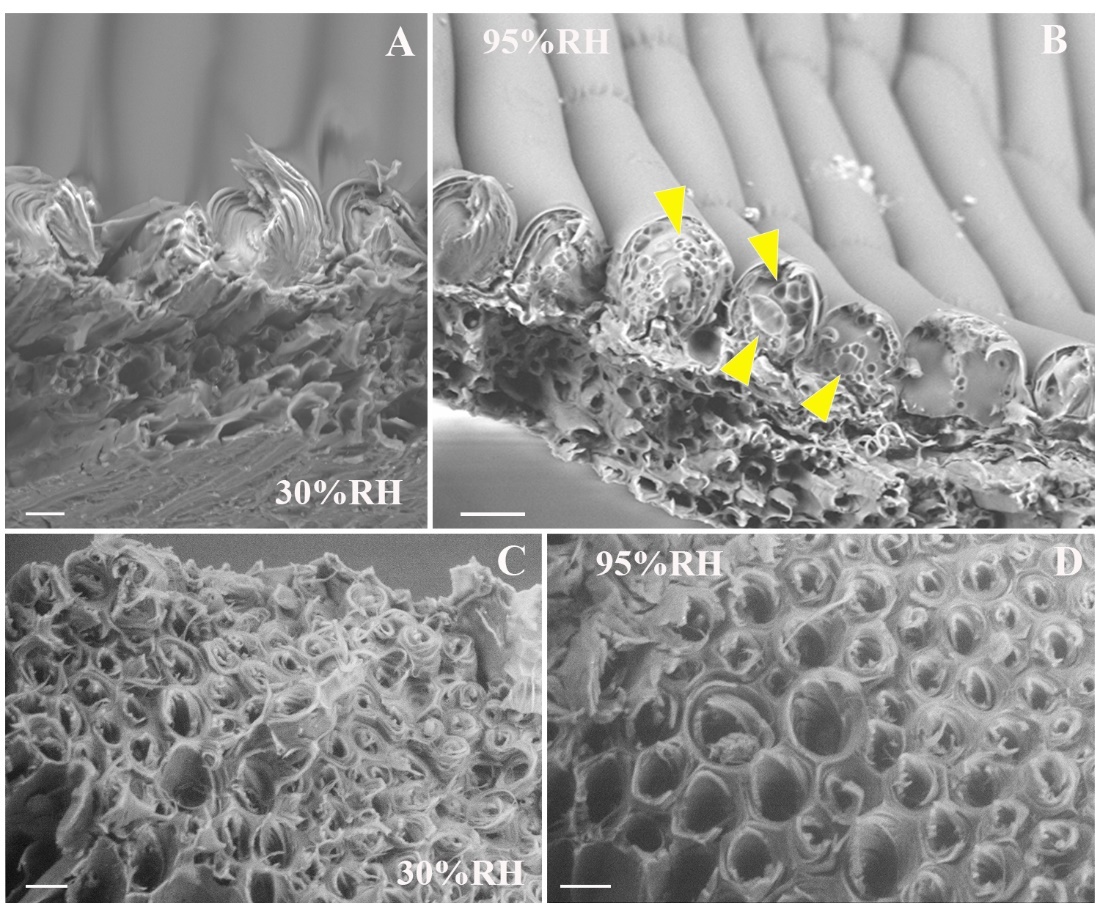


Fig. S5. Hand-broken awn sections of *Pelargonium* and *Erodium* observed with ESEM. A, *Pelargonium* hand-broken section under low relative humidity conditions (30%). B, *Pelargonium* hand-broken section at 100% relative humidity, with yellow arrows indicating the formation of aqueous microbubbles. C, *Erodium* hand-broken section under low relative humidity conditions (30%). D, *Erodium* hand-broken section at 100% relative humidity. The scale bar is 10 µm for (A, C, and D) and 20 µm for (B) (N samples = 3).


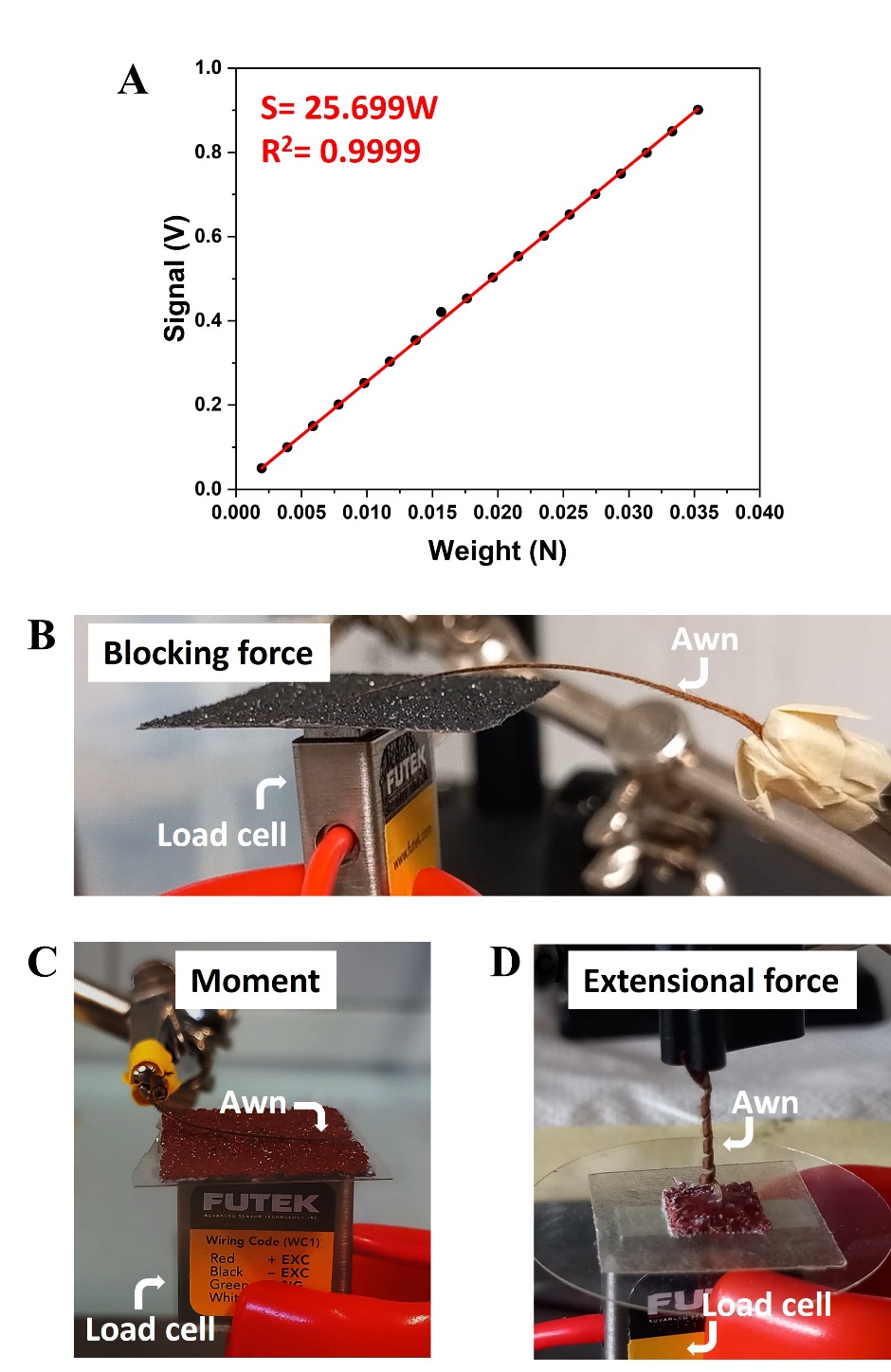


Fig. S6. Load-cell-based setup for blocking force, extensional force and moment measurements, from wet to dry conditions. A, calibration curve. B, blocking force setup. C, moment measurement setup. The moment was calculated considering the length of the awn multiplied by the cosine of the angle between the lever and the orthogonal line to the awn. D, Extensional forces set up.


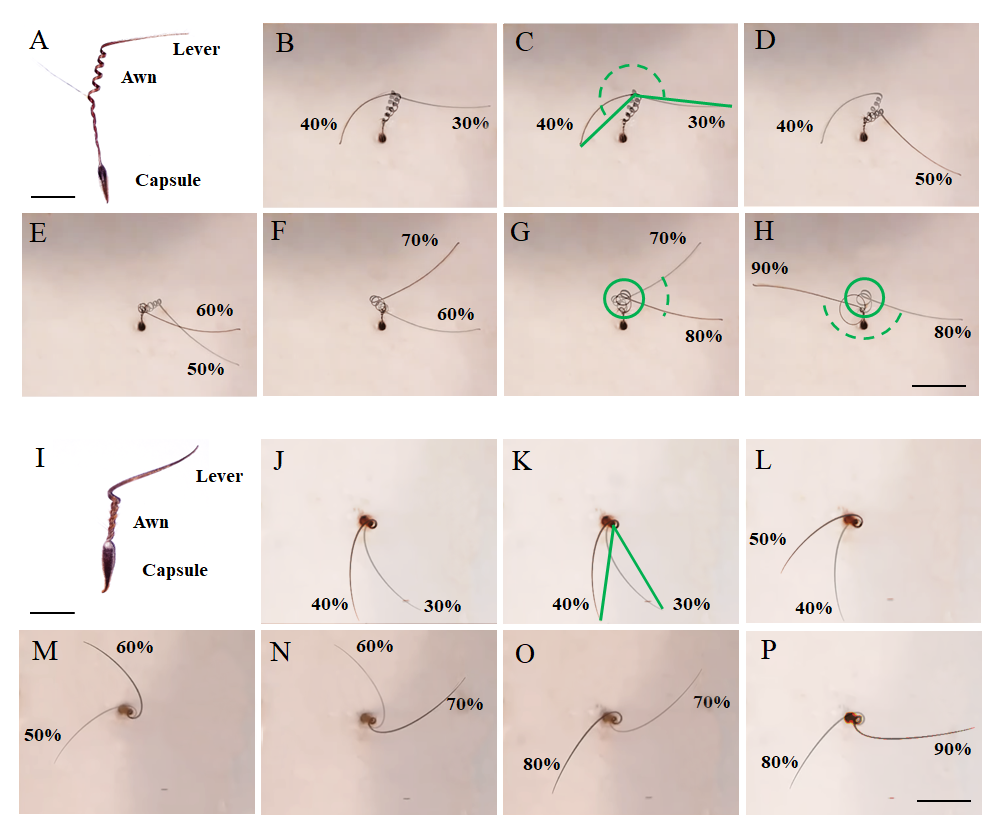


Fig. S7. Angular displacement (θ, °) of *Pelargonium* and *Erodium* vs RH(%). B, superimposed frame of a *Pelargonium* lever at %RH of 30% and 40%. C, an example of angle measurement of the lever rotation from 30% to 40%. D-H, superimposed frame of a *Pelargonium* lever rotation at different %RH: 40%-50% (D), 50-60% (E), 60%-70% (F), 70%-80% (G), 80%-90% (H). I, an image showing the capsule, awn, and lever of *Erodium*. J, superimposed frame of an *Erodium* lever at %RH of 30% and 40%. K, an example of angle measurement of the lever rotation from 30% to 40%. L-P, superimposed frame of an *Erodium* lever rotation at different %RH: 40%-50% (L), 50-60% (M), 60%-70% (N), 70%-80% (O), 80%-90% (P). Scalebar is 1 cm for A, H, I, and P (N samples= 3).


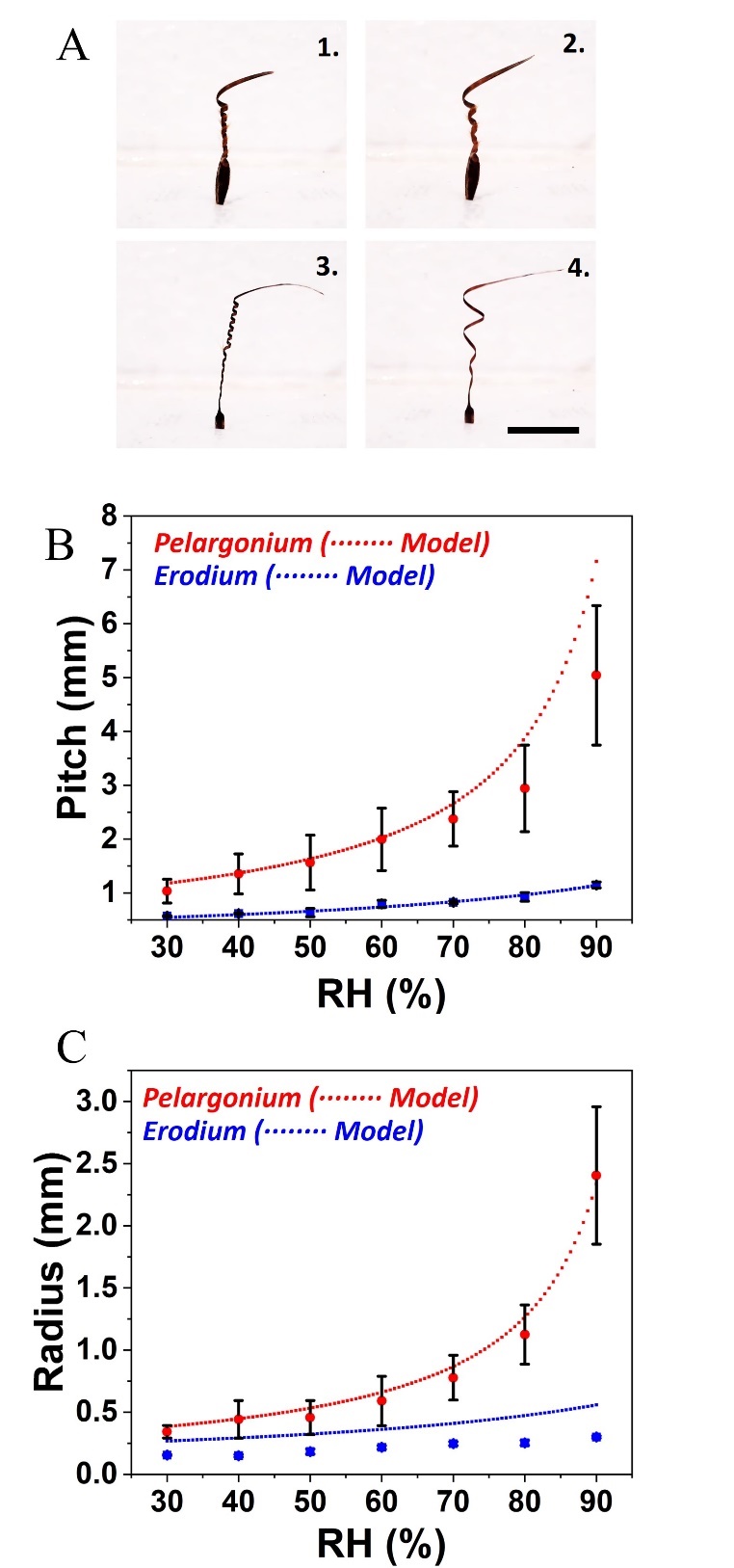


**Fig. S8.** Pitch (P) and Radius (R) for *Pelargonium* and *Erodium* at different RH(%). A, side image of *Pelargonium* at RH= 30% (1) and 90% (2). Side image of *Erodium* at RH= 30% (3) and 90% (4). B, a diagram showing the Radius of *Pelargonium* and *Erodium* at different RH (30-90%). C, a graph showing the Pitch of *Pelargonium* and *Erodium* at different RH (30-90%). The scale bar is 1 cm (N samples= 3). For B and C, the *Pelargonium* data is Reproduced and adapted with permission. (Cecchini, L.; Mariani, S.; Ronzan, M.;Mondini, A.; Pugno, N. M.; Mazzolai, B. 4D Printing of Humidity-Driven Seed Inspired Soft Robots. *Adv. Sci.* 2023, 2205146). Copyright 2023, John Wiley and Sons (CC BY 4.0 DEED).


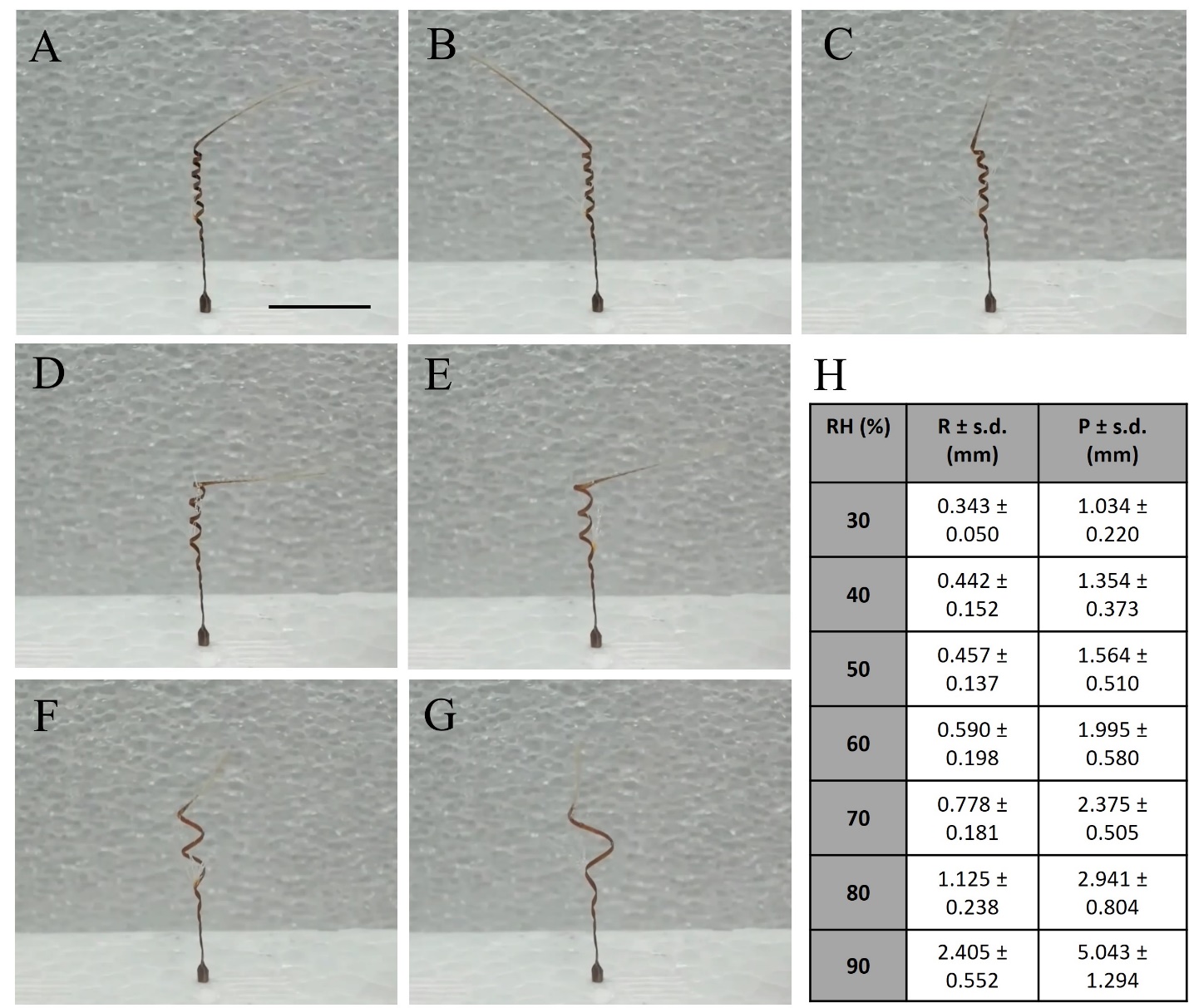


Fig. S9. Data and pictures of P and R for *Pelargonium* at different relative humidity conditions. A, corresponds to a RH of 30%. B, corresponds to a RH of 40%. C, corresponds to a RH of 50%. D, corresponds to a RH of 60%. E, corresponds to a RH of 70%. F, corresponds to a RH of 80%. G, corresponds to a RH of 90%. The scale bar is 1 cm (N samples= 3).


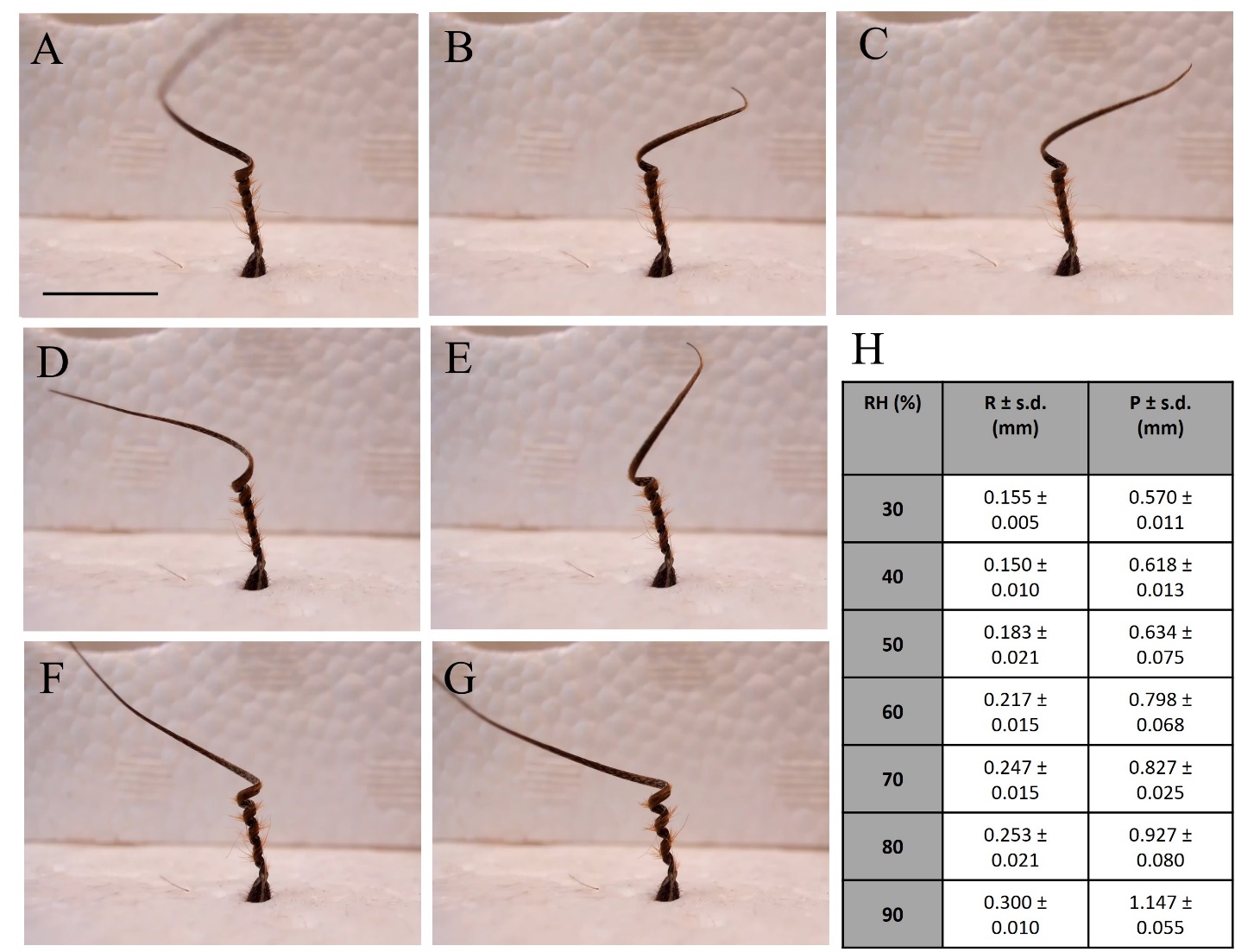


Fig. S10. Data and pictures of P and R for *Erodium* at different relative humidity conditions. A, corresponds to a RH of 30%. B, corresponds to a RH of 40%. C, corresponds to a RH of 50%. D, corresponds to a RH of 60%. E, corresponds to a RH of 70%. F, corresponds to a RH of 80%. G, corresponds to a RH of 90%. The scale bar is 1 cm (N samples= 3).


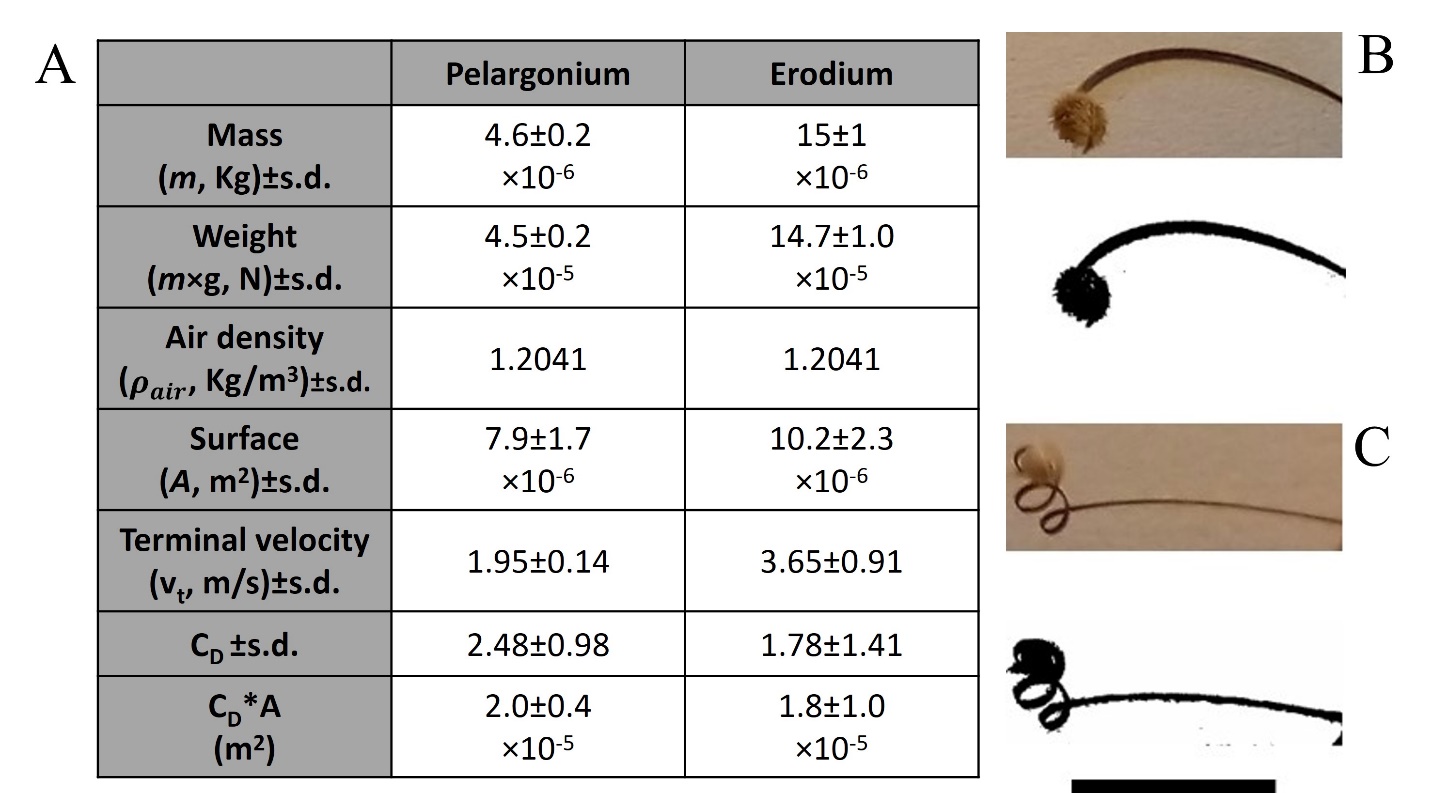


Fig. S11. Aerodynamic performance data of *Pelargonium* and *Erodium*. A, Aerodynamic performance data of *Pelargonium* and *Erodium* during a free fall of 2.0 meters height. B) An *Erodium* binarized image from the top. C) A *Pelargonium* binarized image from the top. The Scale bar is 1 cm, (N samples= 3 for *Pelargonium* and N samples=4 for *Erodium*).


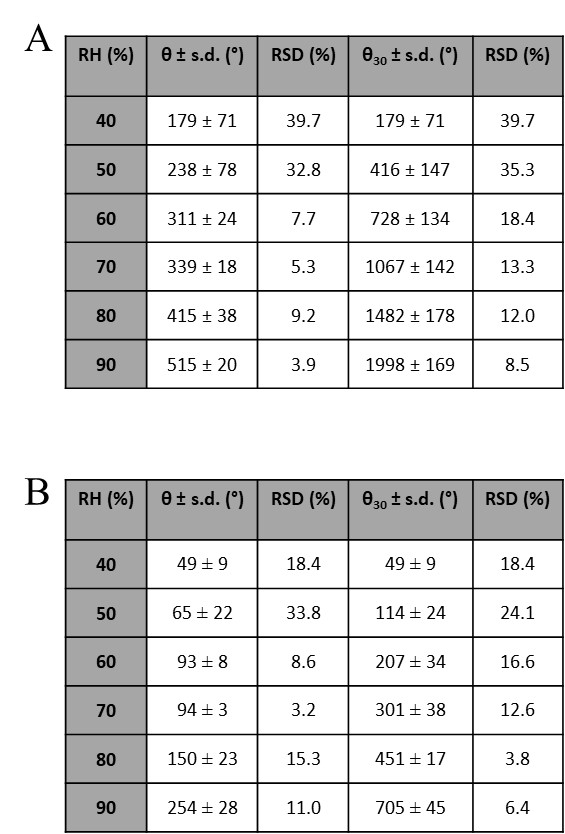


Tables S1. Data of angular displacements. Angular displacement data of *Pelargonium* (A) and *Erodium* (B) lever during relative humidity changes from 30% RH to 90% RH.

Section S1. Elastic energy formula from Blocking Force

To evaluate the elastic energy stored in tissue during the ballistic launch, at first, we measured the blocking force at the tip of the lever as a function of the variation of environmental relative humidity. According to the experiment, it is possible to model the awn in the experiment as a cantilever beam with a single load at one fixed end. Hence, it is possible to model the elastic force as

$$U_{el}= \frac{1}{2} \int_{0}^{l} \frac{{M(x)}^{2}}{E(x)I(x)}dx$$

Where M is the moment, E is the Young’s modulus, I is the second moment of inertia for a rectangular cross-section. Considering the morphometric and nanoindentation characterization, we assumed that the Young’s modulus could be modeled with the mixture rule as a mean value of the active and passive layer’s modulus, almost constant along the length of the awn. Also, E(x) and I(x) are modeled as constant variables with respect to x.

However, the moment M for a cantilever beam with a single load $F_{B}$(blocking force) applied at the end, is equal to:

$$M\left( x \right)=F_{b} l (1- \frac{x}{l})$$

Where l is the length of the awn, hence, it is possible to solve the integral as:

$$U_{el}= \frac{1}{2} \int_{0}^{l} \frac{{M(x)}^{2}}{E(x)I(x)}dx= \frac{1}{2} \frac{F_{b}^{2}}{E I} \int_{0}^{l} \left( l-x \right)^{2}dx=\frac{1}{6} \frac{F_{b}^{2} l^{3}}{E I}= 2 \frac{F_{b}^{2} l^{3}}{E w h^{3}}$$

All the data used for the evaluation of the elastic energy are reported in Table.1 (elastic modulus) and in the morphometric characterization section.

**Section S2.** Modeling of kinematic and dynamic parameters of *Pelargonium* and *Erodium* awns

To predict the kinematic deformation of the awn, we use the theory of laminate composite plates and the model reported by Ha et al., 2020^13^ and subsequently adapted by us using experimental mechanical parameters^14^. Essentially, we consider the hygroscopic structures as trilayers, where the cap layer drives the deformation through hygroscopic actuation. Conceptually the cap layers are decomposed into two identical and homogeneous sublayers, which differ only in fiber orientation. The helical windings of cellulose microfibrils will determine two microfibril angles (MFA), 𝜃_1_ and 𝜃_2_. The deformation of each active layer is anisotropic as confined by the microfibril angles. Then, the awn can be simply modeled as a trilayer structure having two active layers and one inactive layer (Fig. S12).

**Fig. S12**. A simplified sketch of the awn decomposed into trilayers.θ_1_ is MFA of active layer 1, θ_2_ is MFA of active layer 2. Green line represents the cell axis while blue arrows show directions of hygroscopic expansion perpendicular to microfibrils.

The deformation tendencies of the plates depend on the stress generated by hygroscopic expansion and a simple constitutive relation can be expressed as:

$$\boldsymbol{\sigma}=\sum_{i} \boldsymbol{D}_{\boldsymbol{i}} (\boldsymbol{\epsilon-}{\boldsymbol{\epsilon}_{\boldsymbol{h}}}_{\boldsymbol{i}}\boldsymbol{)}$$

where where i represents the i-th layer, $\mathbf{D}_{\mathbf{i}}$the material stiffness and $\boldsymbol{\epsilon}_{\mathbf{h}}$ the hygroscopic strain associated to the i-th layer, modeling the hygroscopic driving effect as detailed below, $\boldsymbol{\sigma}$ is the stress and $\boldsymbol{\epsilon}$ the strain.

Considering the z as the direction along the thickness of the awn, it is possible to describe the total strain at distance z from the bottom plane ($z_{0}=0$) as $\boldsymbol{\epsilon}(z)=\boldsymbol{\epsilon}_{\boldsymbol{0}}+\boldsymbol{\kappa}z$ under Kirchoff assumption, where $\boldsymbol{\epsilon}_{\boldsymbol{0}}$ is the bottom plane strain and $\boldsymbol{\kappa}$ the curvature. The constitutive relation leads to the matrix equation of forces and moments:

$$\mathbf{F}=\mathbf{A}\epsilon_{0}+\mathbf{B}\kappa$$

$$\mathbf{M}=\mathbf{B}\epsilon_{0}+\mathbf{C} \kappa$$

where $\boldsymbol{F}$ is the force matrix, **M** the moment matrix, **A** the extensional stiffness, **B** the coupling stiffness and **C** the bending stiffness defined as:

$$\mathbf{A}=\sum_{k=1}^{n} \mathbf{D}_{k}\left( z_{k+1}-z_{k} \right)$$

$$\mathbf{B}=\frac{1}{2}\sum_{k=1}^{n} \mathbf{D}_{k}\left( z_{k+1}^{2}-z_{k}^{2} \right)$$

$$\mathbf{C}=\frac{1}{3}\sum_{k=1}^{n} \mathbf{D}_{k}\left( z_{k+1}^{3}-z_{k}^{3} \right)$$

where k is the k-th layer definition and **D** is the stiffness matrix defined as:

$$\mathbf{D}= \mathbf{H}^{\mathbf{-1}}\mathbf{D}^{*}\boldsymbol{R H} \boldsymbol{R}^{\boldsymbol{-1}}$$

$$\mathbf{D}^{*}=\frac{E}{1-\nu^{2}}\left( \begin{matrix} 1 & \nu& 0 \\ \nu& 1 & 0 \\ 0 & 0 & \frac{{1-\nu}}{2} \end{matrix} \right)$$

where E is the Young’s modulus, $\nu$ is the Poisson's ratio, and **H** and **R** are respectively transformation matrix and Reuter's matrix, where the cellulose microfibril angle is in relation to the cellulose helix axis $\theta$ (MFAH):

$$\mathbf{H}\boldsymbol{=}\left( \begin{matrix} \mathrm{co}s^{2}\left( \theta\right) & \mathrm{si}n^{2}\left( \theta\right) & 2sin\left( \theta\right)\cos\left( \theta\right) \\ \mathrm{si}n^{2}\left( \theta\right) & \mathrm{co}s^{2}\left( \theta\right) & -2sin\left( \theta\right)\cos\left( \theta\right) \\ -sin\left( \theta\right)\cos\left( \theta\right) & \sin\left( \theta\right)\cos\left( \theta\right) & \mathrm{co}s^{2}\left( \theta\right)-sin^{2}\left( \theta\right) \end{matrix} \right)$$

$$\mathbf{R}\boldsymbol{=}\left( \begin{matrix} 1 & 0 & 0 \\ 0 & 1 & 0 \\ 0 & 0 & 2 \end{matrix} \right)$$

Since **F** and **M** are induced by hygroscopic swelling, the total strain can be modelled according to the definition of hygroscopic expansion coefficient (CHE$),$ that represents the amount of strain generated per percentage unit of relative humidity.

So, the hygroscopic stress can be expressed as $\boldsymbol{\epsilon}_{\boldsymbol{h}}=\boldsymbol{\alpha} \Delta\Phi= \boldsymbol{\alpha} \left( \Phi- \Phi_{0} \right)$, where $\boldsymbol{\alpha}$ is the linear coefficient of hygroscopic expansion, $\Phi$ is the relative humidity and $\Phi_{0}$ is the value of relative humidity at which the mechanical system shows a curvature $\boldsymbol{\kappa}$ = **0**. To calibrate the considered model, $\Phi_{0}$ must be evaluated experimentally (as a working parameter accounting for, e.g., residual stresses and structural imperfections resulting from fabrication). The vectorial CHE is described as:

$$\boldsymbol{\alpha}\mathbf{=}\left( \begin{matrix} \alpha_{xx} \\ \alpha_{yy} \\ \alpha_{xy} \end{matrix} \right)\boldsymbol{= R}\boldsymbol{H}^{\mathbf{-1}}\boldsymbol{R}^{\mathbf{-1}} \left( \begin{matrix} \alpha_{11} \\ \alpha_{22} \\ \alpha_{12} \end{matrix} \right)$$

So, it is possible to represent **F** and **M** considering the definition of hygroscopic strain and supposing that it is the only (internal) force acting on the structure:

$$\mathbf{F}=\int_{0}^{h} \mathbf{D}\boldsymbol{\alpha} \Delta\phi dz$$

$$\mathbf{M}=\int_{0}^{h} \mathbf{D}\boldsymbol{\alpha} \Delta\phi z dz$$

where h is the whole thickness of the n-th layer and$\Delta\phi$ is the relative humidity (RH) variation.

Since material properties are independent of z-axis (neglecting the discontinuity points or considering piecewise-continuous functions such that $d\boldsymbol{D}/dz=0$ and d$\boldsymbol{\alpha}$/dz = 0), we can express **F** and **M** as:

$$\mathbf{F}=\sum_{k=1}^{n} \mathbf{D}_{k}\boldsymbol{\alpha}_{\boldsymbol{k}}\Delta\phi\left( z_{k+1}-z_{k} \right)$$

$$\mathbf{M}=\frac{1}{2}\sum_{k=1}^{n} \mathbf{D}_{k}\boldsymbol{\alpha}_{\boldsymbol{k}}\Delta\phi\left( z_{k+1}^{2}-z_{k}^{2} \right)$$

Now, the strain and the curvature vector are obtained considering the definition of **F** and **M**:

$$\left( \begin{matrix} \boldsymbol{\epsilon}_{\boldsymbol{0}} \\ \boldsymbol{\kappa} \end{matrix} \right)=\left( \begin{matrix} \mathbf{A} & \mathbf{B} \\ \mathbf{B} & \mathbf{C} \end{matrix} \right)^{-1}\left( \begin{matrix} \mathbf{F} \\ \mathbf{M} \end{matrix} \right)$$

The curvature vector $\boldsymbol{\kappa}\boldsymbol{=}\left( \kappa_{xx}\kappa_{yy}\kappa_{xy} \right)^{\boldsymbol{T}}$ can be transformed in the matrix form:

$$\boldsymbol{b} =\left( \begin{matrix} \kappa_{xx} & \kappa_{xy} \\ \kappa_{xy} & \kappa_{yy} \end{matrix} \right)$$

From the Kirchoff’s theory of thin plates, the maximum eigenvalue of the curvature tensor **b** represents the principal curvature $\kappa_{0},$ which determines the helix of shape. The radius R and the pitch P of the helix are finally calculated as:

$$R=\frac{1}{\kappa_{0}}$$

$$P=\frac{2\pi tan\left( \Psi\right)}{\kappa_{0}}$$

where the tilt angle Ψ is the angle between the cellulose helix axis and the cell’s long axis.

Since the structure is subjected to bending and twisting moment, the combined effect give rise to the typical coiled structure described above.

It is now possible to evaluate the force and moment generated by the structure considering the theory of mechanical springs subjected to large deformation. Considering the hygroscopic actuator as a homogeneous helical cylindrical spring with rectangular cross-section, it is possible to evaluate the extensional force F and the moment $M_{0}$ in closed form as:

$$F\left( \phi\right)=\frac{Gk_{s}wh^{3}\cos\beta}{R\left( \phi\right)}\left( \frac{\sin\beta\cos\beta}{R\left( \phi\right)}-\frac{\sin\beta_{0}\cos\beta_{0}}{R_{0}} \right)-\frac{E wh^{3}\sin\beta}{12 R\left( \phi\right)}\left( \frac{\cos^{2} \beta}{R\left( \phi\right)}-\frac{\cos^{2} \beta_{0}}{R_{0}} \right)$$

$$M_{0}\left( \phi\right)=Gk_{s}wh^{3}\sin\beta\left( \frac{\sin\beta\cos\beta}{R\left( \phi\right)}-\frac{\sin\beta_{0}\cos\beta_{0}}{R_{0}} \right)+\frac{Ewh^{3}\cos\beta}{12}\left( \frac{\cos^{2} \beta}{R\left( \phi\right)}-\frac{\cos^{2} \beta_{0}}{R_{0}} \right)$$

Note that in this case we assume that the material can be simplified as a homogeneous structure by means of rule of mixtures, where the material properties of the composite are evaluated considering the volume fraction of the fiber layer with respect to the substrate layer.

Knowing the kinematic variation of the geometrical features involved in hygroscopic coiling process (radius and pitch as function of RH) it is finally possible to estimate in a closed-form solution the dynamic parameters.
